# Genome-Wide Association Studies Reveal All-Stage Rust Resistance Loci in Elite Durum Wheat Genotypes

**DOI:** 10.3389/fpls.2021.640739

**Published:** 2021-04-12

**Authors:** Meriem Aoun, Matthew N. Rouse, James A. Kolmer, Ajay Kumar, Elias M. Elias

**Affiliations:** ^1^Department of Plant Sciences, North Dakota State University, Fargo, ND, United States; ^2^Cereal Disease Laboratory, United States Department of Agriculture–Agricultural Research Service, St. Paul, MN, United States; ^3^Department of Plant Pathology, University of Minnesota, St. Paul, MN, United States

**Keywords:** leaf rust, stripe rust, stem rust, durum wheat, resistance, association mapping, molecular markers

## Abstract

Leaf rust, caused by *Puccinia triticina* (*Pt*), stripe rust caused by *Puccinia striiformis* f. sp. *tritici* (*Pst*), and stem rust caused by *Puccinia graminis* f. sp. *tritici* (*Pgt*) are major diseases to wheat production globally. Host resistance is the most suitable approach to manage these fungal pathogens. We investigated the phenotypic and genotypic structure of resistance to leaf rust, stem rust, and stripe rust pathogen races at the seedling stage in a collection of advanced durum wheat breeding lines and cultivars adapted to Upper Mid-West region of the United States. Phenotypic evaluation showed that the majority of the durum wheat genotypes were susceptible to *Pt* isolates adapted to durum wheat, whereas all the genotypes were resistant to common wheat type-*Pt* isolate. The majority of genotypes were resistant to stripe rust and stem rust pathogen races. The durum panel genotyped using Illumina iSelect 90 K wheat SNP assay was used for genome-wide association mapping (GWAS). The GWAS revealed 64 marker-trait associations (MTAs) representing six leaf rust resistance loci located on chromosome arms 2AS, 2AL, 5BS, 6AL, and 6BL. Two of these loci were identified at the positions of *Lr52* and *Lr64* genes, whereas the remaining loci are most likely novel. A total of 46 MTAs corresponding to four loci located on chromosome arms 1BS, 5BL, and 7BL were associated with stripe rust response. None of these loci correspond to designated stripe rust resistance genes. For stem rust, a total of 260 MTAs, representing 22 loci were identified on chromosome arms 1BL, 2BL, 3AL, 3BL, 4AL, 5AL, 5BL, 6AS, 6AL, 6BL, and 7BL. Four of these loci were located at the positions of known genes/alleles (*Sr7b*, *Sr8155B1*, *Sr13a*, and *Sr13b*). The discovery of known and novel rust resistance genes and their linked SNPs will help diversify rust resistance in durum wheat.

## Introduction

Durum wheat [2n = 4x = 28, AABB, *Triticum turgidum* L. Var. *durum* (Desf.)] is the second most cultivated wheat crop. It accounts for about 8% of the world’s total wheat production (Mengistu and Pè, 2016) and is mainly produced in the Mediterranean region, Eastern Europe, and North America (Royo et al., 2009). Annual worldwide durum wheat production is estimated to be around 36 million tons (Magallanes-López et al., 2017), with approximately, 2.5 million tons produced in the United States. North Dakota’s production of durum wheat accounts for over 50% of total U.S. production (NASS, 2018). Leaf rust, stripe rust and stem are major fungal diseases threatening durum wheat production globally. Rust resistance is the most environmentally and economically feasible approach for managing these diseases. Therefore, the development and deployment of rust resistant cultivars is a major goal of wheat breeding programs worldwide.

Tetraploid durum wheat has historically been more resistant to leaf rust than hexaploid common wheat (*T*. *aestivum* L.) (Singh et al., 2004; Herrera-Foessel et al., 2006). However, during the last 20 years, new durum-type *Pt* races have emerged and caused leaf rust epidemics in several durum wheat producing regions (Singh et al., 2004; Goyeau et al., 2012; Mishra et al., 2015; Aoun et al., 2016). Virulent *Pt* isolates have not been found yet in North Dakota, however, a highly virulent race (BBBQJ) was reported in California and Kansas, United States (Kolmer, 2013, 2015a). This poses a threat to the major durum-producing regions of the USA and Canada. *Pt-*isolates from durum wheat are often avirulent to most leaf rust resistance (*Lr*) genes in common wheat (Huerta-Espino and Roelfs, 1992; Ordoñez and Kolmer, 2007a). The durum wheat type-*Pt* isolates from several durum wheat producing countries have similar phenotypic reactions on ‘Thatcher’ wheat near-isogenic lines and similar or identical SSR and SNP genotypes, suggesting a common origin (Ordoñez and Kolmer, 2007a,b; Aoun et al., 2020; Kolmer et al., 2020). Other *Pt*-isolates collected on tetraploid wheat in Ethiopia (designated as race EEEEE) are avirulent to Thatcher wheat and have a unique molecular genotype compared to all other *Pt*-isolates collected from durum wheat and common wheat globally (Ordoñez and Kolmer, 2007a,b; Kolmer and Acevedo, 2016; Aoun et al., 2020; Kolmer et al., 2020).

A total of 79 *Lr* genes have been identified in wheat, only 20 of them are known to be present in durum wheat (Desiderio et al., 2014; Qureshi et al., 2018). In response to leaf rust epidemics in many durum producing countries, a number of *Lr* genes were identified in this crop including *Lr3a*, *Lr14a*, *Lr27+Lr31*, *Lr61*, *Lr79*, and *LrCamayo* (Herrera-Foessel et al., 2007, 2008a,b; Huerta-Espino et al., 2009; Qureshi et al., 2018). Other not yet cataloged *Lr* genes were also detected in durum wheat landraces and cultivars (Loladze et al., 2014; Aoun et al., 2016, 2017, 2019; Kthiri et al., 2018, 2019). However, due to continuous virulence evolution of *Pt* isolates on many of the identified *Lr* genes, diversifying the genetic basis for leaf rust resistance in durum wheat is a priority.

Stripe rust is another major disease of wheat worldwide (Chen, 2005). Aggressive *Pst* races adapted to high temperatures have emerged and spread into most wheat producing regions (Milus et al., 2009). Over 80 stripe rust resistance (*Yr*) genes have been designated in wheat (McIntosh et al., 2013, 2017, Gessese et al., 2019). The *Yr* genes that were derived from tetraploid wheat (*T. turgidum* L. ssp) include *Yr7*, *Yr15*, *Yr24*/*Yr26*, *Yr30*, *Yr35*, *Yr36, YrH52, Yr53, Yr64*, and *Yr65* (McFadden, 1939; Macer, 1966; McIntosh and Lagudah, 2000; Peng et al., 2000; Ma et al., 2001; Marais et al., 2005; Uauy et al., 2005; Xu et al., 2013; Cheng et al., 2014). However, most of the *Yr* genes identified in wheat are race specific and have become ineffective against the rapidly evolving pathogen (Chen, 2013; McIntosh et al., 2013; Rosewarne et al., 2013). Therefore, identification and pyramiding of new genes is needed for more effective management of this rapidly evolving pathogen.

Stem rust has historically threatened common wheat and durum wheat production. The Ug99 race group that appeared in East Africa overcame several widely used wheat stem rust resistance (*Sr*) genes (Jin et al., 2007; Singh et al., 2011). More than 70 cataloged *Sr* genes have been characterized in durum and common wheat. Only 31 genes are still effective against at least one race of the 13 Ug99 variants (Rouse et al., 2011, 2014; Singh et al., 2011, 2015). Approximately half of these genes were introgressed into wheat from secondary and tertiary gene pools (Rouse et al., 2014; Singh et al., 2015) and only a few genes have been identified in durum wheat Designated *Sr* genes that have be reported in tetraploid wheat include *Sr2*, *Sr7a*, *Sr8b*, *Sr8155B1, Sr9d*, *Sr9e*, *Sr9g*, *Sr11*, *Sr12*, *Sr13a*, *Sr13b*, *Sr14*, and *Sr17* (Jin et al., 2007; Singh et al., 2015; Nirmala et al., 2017; Saini et al., 2018; Zhang et al., 2017).

In North American durum wheat cultivars, resistance to the Ug99 lineage is mainly due to *Sr13*, of which the *Sr13a* allele was first identified in Khapstein, a hexaploid wheat derivative of cultivated emmer wheat (*T. turgidum* L. ssp. *dicoccum*, 2*n* = 4*x* = 28, AABB) cv. Khapli (Knott, 1962; Jin et al., 2007; Klindworth et al., 2007; Zhang et al., 2017). *Sr9e* is also another *Sr* gene frequently deployed in durum wheat (Olivera et al., 2012; Saini et al., 2018). Nirmala et al. (2017) recently identified a possible *Sr8* allele, designated as *Sr8155B1*, in the durum wheat line ‘8155-B1.’ *Sr8155B1* is effective to a variant of the Ug99 race TTKST but not to race TTKSK (Nirmala et al., 2017). However, the frequency of this allele in durum wheat cultivars is not yet determined. Besides the Ug99 race group, additional *Pgt*-races with broad virulence spectra have also emerged during the last decade including TRTTF, JRCQC, and TKTTF. These races do not belong to the Ug99 lineage and pose serious threat to common wheat and durum production (Olivera et al., 2012, 2015). Among these races, TRTTF and JRCQC were reported to be virulent to the major known components of stem rust resistance in North American durum cultivars *Sr13* and *Sr9e* (Olivera et al., 2012). However, according to Zhang et al. (2017), *Sr13a* is effective to both JRCQC and TRTTF, and *Sr13b* is effective to TRTTF, but not JRCQC. Identifying and characterizing new sources of stem rust resistance in durum wheat is needed to manage future outbreaks.

This study was designed: (1) to determine levels of leaf rust, stem rust, and stripe rust resistance in a large collection of elite durum wheat lines at seedling stage, (2) to determine the genetic architecture of rust resistance loci using GWAS and Infinium 90K wheat SNP assay (3) to detect novel seedling resistance (all-stage resistance) loci to *Pt*, *Pst*, and *Pgt* races that could be used in breeding programs, and (4) to identify SNPs associated with seedling rust resistance loci for marker assisted breeding.

## Materials and Methods

### Plant Materials

A collection of 248 durum wheat genotypes was used in this study. The collection represented advanced breeding lines evaluated in the North Dakota State University’s (NDSU) Uniform Regional Durum Nursery (URDN) from 1997 to 2014 (for more details, see Johnson et al., 2019; Supplementary Table 1). These genotypes were regularly evaluated for agronomic and quality traits over the years in multiple environments. Thus, this plant material represents the core of the NDSU’s durum breeding program.

### Leaf Rust Phenotyping

The durum wheat collection was screened at the seedling stage with six *Pt* isolates (Supplementary Table 1). Five of these isolates (TUN 20-1, ETH 13D17-1, MEX10, ETH 63-1, and MOR 33-1) were durum wheat type isolates, while ALK-ND is a common wheat type isolate from North Dakota. The virulence/avirulence phenotypes of the *Pt* isolates were based on the infection types (ITs) of 20 Thatcher near-isogenic lines (NILs) at seedling stage as described by Long and Kolmer (1989). The Tunisian (TUN 20-1) and Moroccan (MOR 33-1) isolates were both of race BBBSJ (virulent to the *Lr* genes *LrB*, *Lr10*, *Lr14a, Lr14b*, and *Lr20*). The Mexican isolate MEX10 was of race BBBQJ (virulent to the *Lr* genes *LrB*, *Lr10*, *Lr14b*, and *Lr20*). The two Ethiopian isolates ETH 63-1 and ETH 13D17-1 designated as race EEEEE are avirulent on the Thatcher wheat. The common wheat type isolate ALK-ND, designated as race MBDSS was isolated from the durum wheat cultivar ‘Alkabo’ (PI 642020) in North Dakota and is virulent to the *Lr* genes *Lr1*, *Lr3a*, *Lr3bg*, *Lr10, Lr14a*, *Lr14b*, *Lr17*, *Lr20*, and *LrB*.

The phenotyping using isolates EEEEE_ETH 63-1, BBBSJ_MOR 33-1, and MBDSS_ALK-ND was performed at the biosafety level-2 facility at the Agricultural Experiment Station Greenhouse Complex, Fargo, ND, United States using a randomized complete block design (RCBD) with two replicates. In each replicate five-to-seven plants/line were tested and the common wheat cultivar Thatcher and the leaf rust susceptible durum line ‘RL6089’ were included twice as checks in each of the 50-cell trays. For each experiment, two replicates of Thatcher NIL differentials were included to confirm the virulence phenotype of *Pt*-isolates. Seedling growth conditions, inoculum increase and preparation, inoculation, and greenhouse conditions under which the inoculated plants were kept until disease phenotyping were as described by Aoun et al. (2019).

The screening experiments with the remaining three isolates EEEEE_ETH 13D17-1, BBBQJ_MEX10, and BBBSJ_TUN 20-1 were done at the U.S. Department of Agriculture- Agricultural Research Service (USDA–ARS), Cereal Disease Laboratory (CDL) in Saint Paul, MN, United States. The seedling tests using these three isolates were performed in a single replicate with five-to-seven plants/line. The common wheat Thatcher and the durum line RL6089 were included as checks. The detailed protocols of plant growing conditions and inoculation were described in Kolmer and Hughes (2013).

Leaf rust ITs were taken 12–14 days after inoculation on the second leaf using a 0–4 scale (Long and Kolmer, 1989; McIntosh et al., 1995) where IT of ‘0’ = no visible symptoms, IT of ‘;’ = hypersensitive flecks, IT of ‘1’ = small uredinia with necrosis, IT of ‘2’ = small-to medium-size uredinia surrounded by chlorosis, IT of ‘3’ = medium-size uredinia with no chlorosis or necrosis, and IT of ‘4’ = large uredinia with no necrosis or chlorosis. Larger and smaller uredinia than expected for each IT were designated with ‘+’ and ‘–‘, respectively. Seedling plants exhibiting ITs of 0–2^+^ and ‘X’ (a mixture of resistant and susceptible reactions evenly distributed on the leaf surface) were considered resistant, whereas seedling plants with ITs of 3–4 were considered susceptible (Long and Kolmer, 1989; McIntosh et al., 1995). In situations where multiple ITs were observed on the same leaf surface, the plant reaction was recorded as the most predominant IT followed by the least predominant IT.

### Stripe Rust Phenotyping

Three *Pst* races (PSTv-37, PSTv-41, and PSTv-52) collected from common wheat in North Dakota (Supplementary Table 2) were used to screen the durum genotypes. These three *Pst* races are the only ones currently present in North Dakota. PSTv-37 has been the most widely distributed race across the United States (Wan et al., 2016) and has a virulence/avirulence phenotype of *Yr6*, *7*, *8*, *9*, *17*, *27*, *43*, *44*, *Tr1*, *Exp2*/*Yr1*, *5*, *10*, *15*, *24*, *32*, *SP*, *76.* The race PSTv-52 that has been widely distributed in the United States^1^ has a virulence/avirulence profile of *Yr6*, *7*, *8*, *9*, *17*, *27*, *43*, *44*, *Exp2*/*Yr1*, *5*, *10*, *15*, *24*, *32*, *SP*, *Tr1*, *76.* The race PSTv-41 is considered the most virulent race in ND and has a virulence/avirulence profile of *Yr6*, *7*, *8*, *9*, *10*, *17*, *24*, *27*, *32*, *43*, *44*, *Tr1, Exp2*/*Yr1*, *5*, *15*, *SP*, *76.*

To screen for stripe rust, three separate experiments (one experiment/*Pst* race) with the same set of durum genotypes (*n* = 248) were planted at the Fargo Agricultural Experiment Station Greenhouse Complex, ND, United States. In each experiment, five-to-seven seeds/genotype were planted in 50-well trays. The susceptible cultivar ‘Avocet’ was included twice in each tray as check. To confirm the race identity, a set of 18 differential lines containing each a single *Yr* gene (Wan and Chen, 2014) was included alongside each single-race experiment. The seedlings were grown in a rust-free greenhouse at 22°C/18°C (day/night) and 16 h photoperiod. When the second leaves were fully expanded, the plants were spray inoculated with fresh rust urediniospores suspended in Soltrol-170 oil (Phillips Petroleum, Bartlesville, OK, United States) at a concentration of 0.01 g/mL. After the Soltrol-170 oil dried on the leaf surface, the inoculated plants were incubated in a dark dew chamber at 10°C with 100% relative humidity for 24 h. The seedlings were later transferred to a rust-free incubated growth chamber at 17°C/8°C (day/night) and 16 h photoperiod. The seedling ITs were recoded 16–18 days post-inoculation on a scale of 0–9 (Line and Qayoum, 1992). IT of ‘0’ = no visible signs or symptoms, IT of ‘1’ = necrotic or chlorotic flecks with no sporulation; IT of ‘2’ = necrotic and/or chlorotic blotches or stripes with no sporulation; IT of ‘3’ = necrotic and/or chlorotic blotches or stripes with only a trace of sporulation; IT of ‘4,’ ‘5,’ and ‘6’ corresponds to necrotic and/or chlorotic blotches or stripes with light, intermediate, and moderate sporulation, respectively; and IT of ‘7,’ ‘8,’ and ‘9’ corresponds to abundant sporulation with necrotic and/or chlorotic stripes or blotches, chlorosis around the sporulation area, and no chlorosis or necrosis, respectively. ITs from 0 to 3 were considered resistant responses, ITs from 4 to 6 were considered intermediate responses and ITs from 7 to 9 were considered susceptible responses.

### Stem Rust Phenotyping

The durum wheat genotypes were tested at seedling stage with six African *Pgt* races TTKSK (isolate 04KEN156/04; Jin and Singh, 2006), TTKST (06KEN19v3; Jin et al., 2008), TTKTT (14KEN58-1; Newcomb et al., 2016), TKTTF (13ETH18-1; Olivera et al., 2015), TRTTF (06YEM34-1; Olivera et al., 2012), and JRCQC (09ETH08-3; Olivera et al., 2012) (Supplementary Table 3). The durum lines were phenotyped in the biosafety level-3 facility at the USDA-ARS CDL in St. Paul, MN, United States. The lines were planted in two replicates corresponding to different experiments with different planting and inoculation dates. Five seedlings per line were planted per replicate for all six *Pgt* races. The inoculum preparation, inoculation, greenhouse conditions, and disease screening were as described by Hundie et al. (2019). In brief, the urediniospores stored at –80°C were heat shocked at 45°C for 15 min. For inoculation, gelatin capsules including 14 mg spores were suspended in 0.75 ml mineral oil (Sotrol 170, Phillips Petroleum, Borger, TX, United States) and sprayed onto the plant primary leaves of 240 wheat seedling plants corresponding to 48 wheat lines. After the Soltrol-170 oil dried on the leaf surface, the inoculated plants were placed in a humidity chamber in the dark for 14-to-16 h at 22°C, then exposed to high pressure sodium vapor lamps for 3–4 h. The plants were then transferred to the greenhouse and kept at temperature of 19–22°C and 16 h photoperiod for 10–12 days. The seedling ITs were scored using the Stakman 0–4 scale (Stakman et al., 1962). Plants with ITs of 0–2^+^3 were considered resistant and those with IT of 3–4 were considered susceptible.

### Phenotypic Data Analysis

For statistical analysis, the 0–4 scale for leaf rust and stem rust screening was converted to a linearized 0–9 scale (Zhang et al., 2014) where plants with ITs of 0–6 were classified as resistant and those with ITs of 7–9 were considered susceptible. For further analysis, the mean of replicates per trait were used. Pairwise Pearson’s correlations between traits were calculated and plotted using the ‘corrplot’ package (Wei and Simko, 2013) in the software R 3.4.1 (R Core Team, 2016). Correlation values were considered significantly different from zero at *P*-value ≤ 0.05.

### Genotyping

The durum collection was genotyped as described by Johnson et al. (2019) using the Illumina iSelect 90K wheat SNP assay (Wang et al., 2014). The 90K wheat SNP assay generated 17,377 polymorphic SNPs. Markers which were in common with those included in the tetraploid wheat consensus map (Maccaferri et al., 2015) were kept for further analysis (Supplementary Table 4). Additionally, a diagnostic marker for the presence of either *Sr13* allele (Zhang et al., 2017), a linked marker to *Sr8155B1* (Nirmala et al., 2017), and three dCAPS markers used to discriminate *Sr13a* and *Sr13b* were also used to genotype the durum wheat collection. The durum wheat collection was genotyped using derived cleaved amplified polymorphic sequence (dCAPS) markers for *Sr13* and its three alleles R1 (*Sr13a-R1*), R2 (*Sr13b*), and R3 (*Sr13a-R3*). Markers *dCAPS_Sr13* (Zhang et al., 2017), *dCAPS_Sr13_R1cut*, *dCAPS_Sr13_R2nocut*, and *dCAPS_Sr13_R3nocut* were used to identify *Sr13*, *Sr13a-R1*, *Sr13b*, *Sr13a-R3*, respectively (Supplementary Table 5). *Sr13a-R1* and *Sr13a-R3* correspond to the two resistant haplotypes of *Sr13a*: R1 and R3 (Zhang et al., 2017). The dCAPs markers used to discriminate among the two *Sr13* alleles were designed based on the sequence information of the resistant haplotypes of *Sr13* in Zhang et al. (2017). The primer sequences of *Sr13* gene/alleles, the restriction enzymes (RE), and the resulting PCR product sizes after RE digestion are described in Supplementary Table 5. The Kompetitive Allele Specific PCR (KASP) marker (*KASP_6AS_IWB10558*) was used to postulate the presence of the gene *Sr8155B1* (Nirmala et al., 2017). Heterozygotes were converted into missing data. Polymorphic markers with >10% missing data and minor allele frequency (MAF) < 3% were excluded from further analysis.

### Linkage Disequilibrium and Population Structure

Linkage disequilibrium (LD) was performed using JMP Genomics 8.1 software (SAS Institute Inc, 2004) as described by Aoun et al. (2016). The LD estimates for intrachromosomal markers were calculated as the squared correlation coefficient (*R*^2^) for each of the marker pairs. The genome-wide LD decay was estimated by plotting LD estimates (*R*^2^) from all 14 durum wheat chromosomes against the corresponding pairwise genetic distances in cM. The genetic positions of the markers were according to the durum wheat consensus map of Maccaferri et al. (2015). Smoothing spline fit was applied to LD decay plot.

The principal component analysis (PCA) was used to examine the population structure (Q matrix). SNPs with LD (*R*^2^) ≤ 0.2 were used to estimate the Q matrix. The identity-by-state (IBS) matrix or Kinship matrix (K matrix) that represents the proportion of shared alleles for all pairwise comparisons between genotypes was also estimated. The K and Q matrices were estimated using JMP Genomics 8.1 software.

### Genome-Wide Association Analysis

For each trait, mixed linear model for genome-wide association analyses were performed using JMP Genomics 8.1 software. Five regression models were tested to identify the best model per trait from which MTAs will be derived. The tested models include (i) naïve, (ii) kinship, (iii) kinship plus population structure (first two PCs), (iv) kinship plus population structure (first three PCs), and (v) kinship plus population structure (first four PCs). The K and the Q matrices were included in the genome-wide association analysis model to reduce the chance of false-positive MTAs. Each of the markers was fitted into the regression equation to generate a *P*-value. The best association mapping model (of the five tested regression models) was selected based on the Bayesian Information Criterion (BIC), where the lowest BIC value corresponded to the best model (Ghosh et al., 2006; Zhang et al., 2010). For each trait, the marker *P*-values of the selected model were used to calculate the *P*-value of the false positive discovery rate (FDR) (Benjamini and Yekutieli, 2001). MTAs were considered significant at *P*-value of FDR ≤ 0.05. The LD estimates between significant markers and marker genetic positions on the tetraploid consensus map (Maccaferri et al., 2015) were used to group MTAs from the GWAS into the same or different underlying loci. Each locus was represented by the most significant SNP marker. The physical and genetic position of the most significant marker per locus and any markers from the literature used for comparative mapping was based on the durum wheat cv. Svevo genome v1 (Maccaferri et al., 2019) and the tetraploid consensus map (Maccaferri et al., 2015), respectively. In the case of multiple identified loci on the same chromosome, the loci were ordered according to their most significant SNP genetic positions on the tetraploid consensus map of Maccaferri et al. (2015).

## Results

### Phenotypic Data

#### Leaf Rust

**FIGURE 1:**
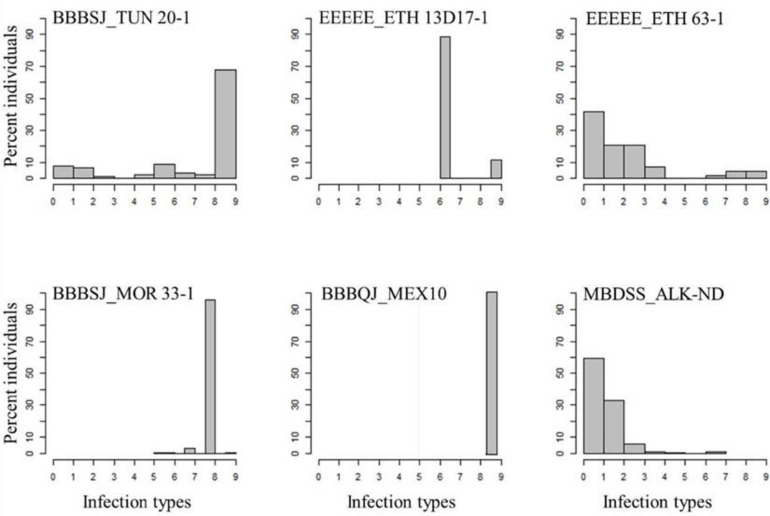
Distributions of the seedling responses of the durum wheat genotypes to *Puccinia triticina* isolates BBBSJ_TUN 20-1, EEEEE_ETH 13D17-1, EEEEE_ETH 63-1, BBBSJ_MOR 33-1, BBBQJ_MEX10, and MBDSS_ALK-ND. X-axis corresponds to linearized Stakman scale (0-to-9).

All the durum wheat genotypes were resistant to the common wheat type isolate MBDSS_ALK-ND. For the *Pt* durum wheat type isolates, the percentage of susceptible lines varied depending on the isolate (Supplementary Table 1). For instance, 10% of the genotypes were susceptible to the Ethiopian isolate EEEEE_ETH 63-1, while 28% of the genotypes showed susceptibility to the Ethiopian isolate EEEEE_13D17-1. The distribution of the ITs to EEEEE_13D17-1 was bimodal, where two ITs were observed. A total of 72% of the genotypes exhibited a mesothetic reaction (IT = ‘3^+^;’), while the remaining genotypes showed IT = ‘3^+^’. The plant reactions to EEEEE_ETH 63-1 ranged between ‘;’ and 3^+^. Even though the two Ethiopian isolates had similar race designation EEEEE (avirulent to the common wheat cv. Thatcher), they carried different virulence/avirulence phenotypes to the durum genotypes in our study (Figure 1 and Supplementary Table 1).

In contrast to the Ethiopian isolates, the percentages of susceptible genotypes to durum wheat type isolates from Morocco, Tunisia, and Mexico were much higher. For instance, all the durum genotypes were susceptible to isolate BBBQJ_MEX10. Similarly, 74 and 98% of the genotypes were susceptible to isolates BBBSJ_TUN 20-1 and BBBSJ_MOR 33-1, respectively. The most resistant lines to race BBBSJ_MOR 33-1 had IT of ‘23,’ whereas the most resistant lines to race BBBSJ_TUN 20-1 had IT of ‘;’suggesting that these two isolates of the same race (based on Thatcher wheat differentials) carried different virulence/avirulence profiles to durum wheat (Figure 1 and Supplementary Table 1).

The top four durum wheat cultivars grown in ND in 2019 were Joppa (PI 673106, 30.2%), Divide (PI 642021, 21.2%), Alkabo (PI 642020, 7.8%), and Carpio (PI 670039, 6.1%) (USDA, NASS, North Dakota Field Office, 2019). All of these cultivars were resistant to EEEEE_ETH 13D17-1, EEEEE_ETH 63-1, and MBDSS_ALK-ND but susceptible to the Mexican isolate BBBQJ_MEX10. Joppa showed intermediate IT to BBBSJ_TUN 20-1 (IT = ‘23’) and to BBBSJ_MOR 33-1 (IT = ‘32^+^′). Divide was resistant to BBBSJ_TUN 20-1, whereas Alkabo and Carpio were susceptible to this Tunisian isolate. Divide was resistant to BBBSJ_MOR 33-1, while Alkabo and Carpio were susceptible to this isolate (Supplementary Table 1).

#### Stripe Rust

**FIGURE 2:**
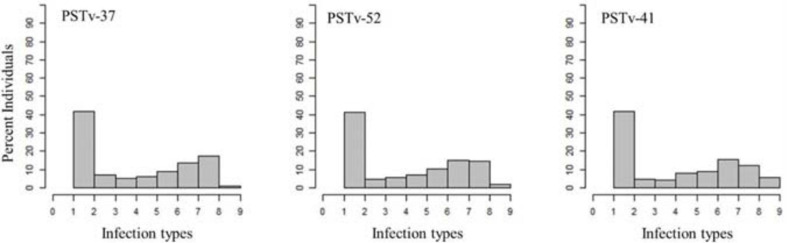
Distributions of the seedling responses of the durum wheat genotypes to *Puccinia striiformis* f. sp. *tritici* races PSTv-37, PSTv-41, and PSTv-52.

A total of 69% of the durum wheat genotypes were resistant to races PSTv-37 and PSTv-52, while 67% of the lines were resistant to race PSTv-41. The ITs to the three *Pst* races ranged between 1 and 9. The cultivars Divide, Alkabo, Carpio were resistant to all the three *Pst* races. Joppa was resistant to races PSTv-37 and PSTv-41 but not to PSTv-52 (Figure 2 and Supplementary Table 2).

#### Stem Rust

**FIGURE 3:**
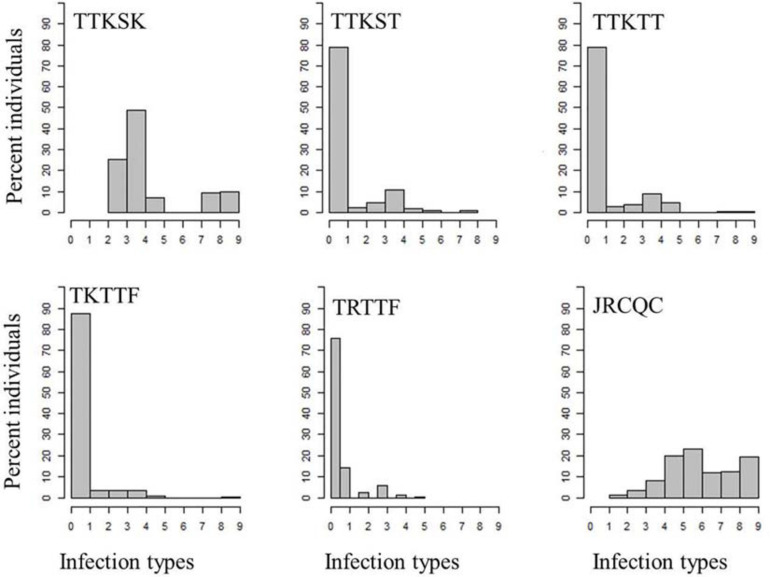
Distributions of the seedling responses of the durum wheat genotypes to *Puccinia graminis* f. sp. *tritici* races TTKSK, TTKST, TTKTT, TKTTF, TRTTF, and JRCQC. X- axis corresponds to the linearized Stakman scale (0-to-9).

About 81–99% of the genotypes were resistant to the three Ug99-lineage races TTKSK, TTKST, and TTKTT. For race TTKSK, the ITs ranged from 1 to 3^+^ with most of the lines showing IT of ‘2^–^’. The ITs to races TTKST and TTKTT ranged between 0; and 33^+^ with most of the genotypes showing IT = ‘0;’. For race TKTTF, only the breeding line ‘D07726’ showed a susceptible IT, while the remaining genotypes showed resistant ITs that ranged between ‘0;’ and ‘2’ with the most frequent resistant IT = ‘0;’ (Figure 3 and Supplementary Table 3).

All the durum wheat genotypes showed resistant ITs to race TRTTF ranging between ‘0;’ and ‘2.’ Like races TTKST, TTKTT, and TKTTF, the most frequent resistant IT to race TRTTF was ‘0;’. Even though, 99–100% of the durum genotypes were resistant to race TTKST, TTKTT, TKTTF, and TRTTF, there were phenotypic variations within the resistant ITs (Figure 3 and Supplementary Table 3) appropriate to conduct further analysis (e.g., GWAS). Of all the *Pgt* races used for screening, race JRCQC was the most virulent race on the durum wheat collection, with 44% of the genotypes showing susceptibility. The resistant ITs to JRCQC ranged from ‘1;’ to ‘2^+^3’ with most of the resistant genotypes showing ITs of ‘22^+^’ to ‘2^+^3’ (Figure 3 and Supplementary Table 3). The durum cultivars Carpio and Alkabo showed resistance to all *Pgt-*races. Divide was resistant to all races except TTKSK and JRCQC, while Joppa was resistant to all races except TTKSK (Supplementary Table 3).

#### Phenotypic Data Correlations

**FIGURE 4:**
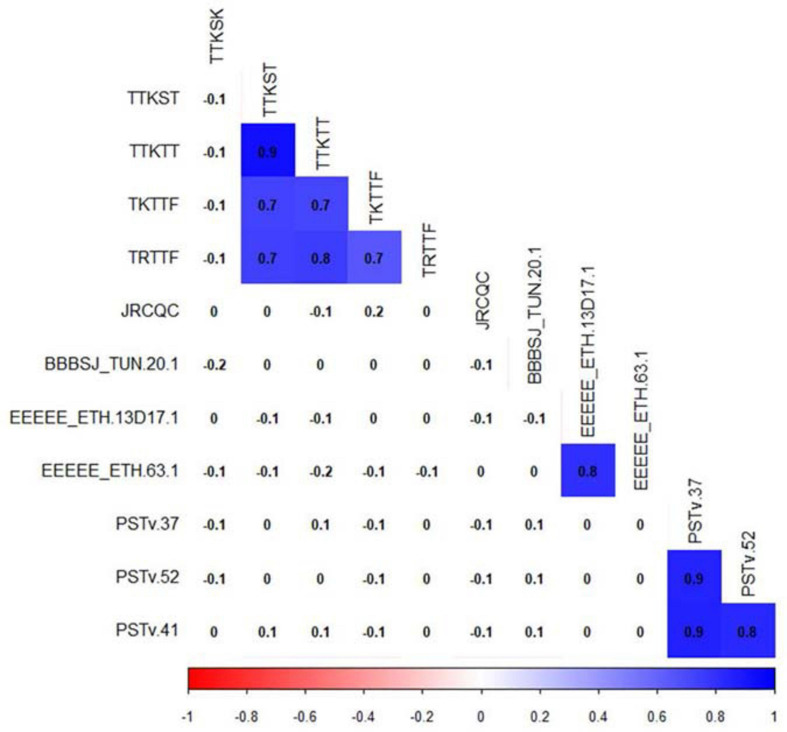
Correlation between durum wheat line infection types to leaf rust, stem rust, and stripe rust pathogen races. Cells with significant correlations at *P*-value < 0.05 were in blue color.

For correlation analyses, we considered only traits with phenotypic variations (Figure 4). Pearson’s correlation between linearized ITs showed a significant correlation (*r* = 0.8, *P*-value ≤ 0.05) between the durum genotype responses to the Ethiopian *Pt* races EEEEE_ETH 63-1 and EEEEE_13D17-1. However, there were no significant correlations between the ITs to BBBSJ_TUN 20-1 and the ITs to both Ethiopian isolates of race EEEEE. There were strong significant correlations between ITs to the three *Pst* races that ranged between 0.8 and 0.9. For *Pgt* races, we observed significant correlations (*r* = 0.7–0.9, *P*-value ≤ 0.05) between ITs to races TTKST, TTKTT, TKTTF, and TRTTF. ITs to TTKSK and JRCQC were not significantly correlated with ITs to any of the remaining four *Pgt-*races. There was no correlation between ITs to TTKSK and JRCQC. We found no significant correlations between ITs to different rust pathogens, suggesting that different genetic loci confer resistance to leaf rust, stripe rust, and stem rust in this durum wheat collection (Figure 4).

### Marker Properties and Linkage Disequilibrium Analysis

**FIGURE 5:**
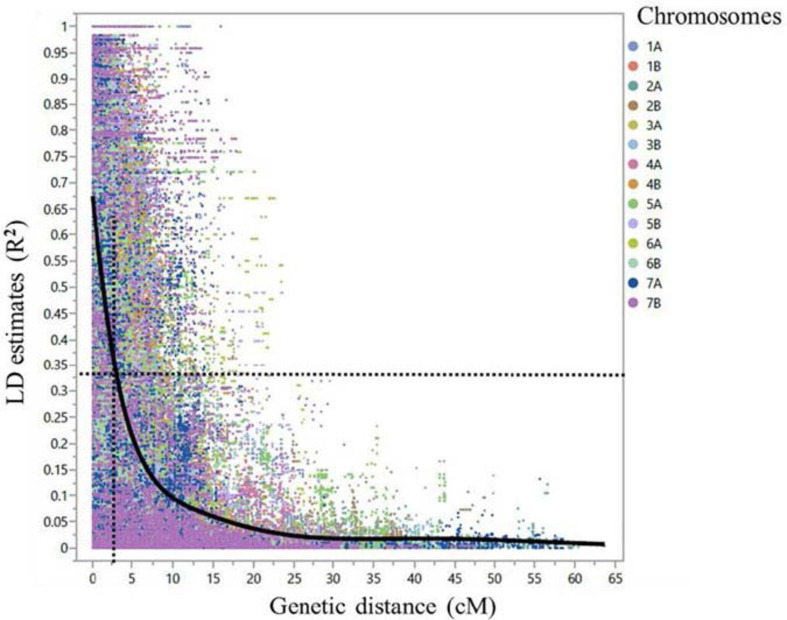
Scatter plot demonstrating linkage disequilibrium (LD) decay across the 14 durum wheat chromosomes for the 248 durum wheat genotypes. The LD estimate (*R*^2^) for pairs of SNPs was plotted against the corresponding genetic distance in centimorgan (cM) based on the tetraploid consensus map of Maccaferri et al. (2015). The dashed lines represent the LD decay that dropped by half at around 2.5 cM in average.

After marker filtering, 10,891 SNPs included in the tetraploid wheat consensus map with MAF ≥ 3% and missing data points ≤ 10% were used for further analysis. Of the 10,891 SNPs, there were 4,779 (43.9%) SNPs on the genome A and 6,112 (56.1%) SNPs on the genome B. Additional four diagnostic dCAPS markers for *Sr13* gene/alleles and a single KASP marker for *Sr8155B1* gene were included. The genome-wide linkage disequilibrium (LD) dropped by half to 0.33 within 2.5 cM on average (Figure 5). Therefore, MTAs from the GWAS within 2.5 cM on average and with LD (*R*^2^) ≥ 0.3 were considered underlying the same locus. In addition, we considered the pairwise LD (*R*^2^ cutoff = 0.3) between significant markers on the same chromosome arm to identify the loci.

### Kinship Analysis, Population Structure, and Regression Model Selection for GWAS

For the identity-by-state matrix or kinship matrix (*K* matrix), there were some hotspots (red color in the heat map) between some of the durum genotypes (Supplementary Figure 1). This suggests intermediate familial relatedness between genotypes as described by Johnson et al. (2019). The PCA showed that the first two, three, four, and 10 PCs explained a cumulative variance of 9.4, 13.2, 16.5, and 31.4% of variation, respectively. The genotypes were clustered into three groups (Johnson et al., 2019) with majority of the lines grouped within the same cluster (Supplementary Figure 2). This is expected because the genotypes are from the same breeding program. Based on BIC values, mixed linear models that include both *Q* and *K* matrices were used for the GWAS for most traits. For traits associated with responses to *Pgt* races TTKST and TKTTF, the best GWAS regression models included the *K* matrix but not the *Q* matrix (Table 1).

**TABLE 1:** Bayesian Information Criterion of association mapping models for each trait.

**Rust trait**	** Races/isolates**	**Naive**	**Kinship**	**2PCs + Kinship^a^**	**3PCs + Kinship^b^**	**4 PCs + Kinship^c^**
Leaf rust	BBBSJ _TUN 20-1	1206.9	1195.9	1183.5	1180.6	**1175.6^d^**
	EEEEE_ETH 13D17-1	683.5	672.4	671.1	661	**658**
	EEEEE_ETH 63-1	1088.5	1077.5	1074.1	1057.6	**1049.3**
Stripe rust	PSTv-41	1204.8	1193.8	1180.9	**1124**	1173.3
	PSTv-52	1158.3	1063.2	1131.1	**1123.8**	1120.5
	PSTv-37	1180.6	1169.6	1159.2	1152.3	**1150.8**
Stem rust	TTKSK	1023.2	935.4	**935.3**	955.7	955.7
	TTKST	948.5	**752.9**	893.9	869.3	867.6
	TTKTT	957.6	752.9	**749.7**	752.9	889.3
	TKTTF	796.5	**666.5**	702.5	686.4	685.0
	TRTTF	663.9	652.8	611.5	600.7	**599.7**
	JRCQC	1002	864.2	863.4	863.2	**853.2**

*^*a*^2PC, population structure matrix (*Q* matrix) based on the first two principal components explaining 9.4% of variation. ^*b*^3PC, population structure matrix (*Q* matrix) based on the first three principal components explaining 13.2% of variation. ^*c*^4PC, population structure matrix (*Q* matrix) based on the first four principal components explaining 16.5% of variation. ^*d*^Numbers in bold indicate the lowest Bayesian Information Criterion that corresponds to the best regression model for each trait. The best model was used to investigate marker-trait associations.*

### Marker–Trait Associations

#### Association Analysis for Leaf Rust Response

**FIGURE 6:**
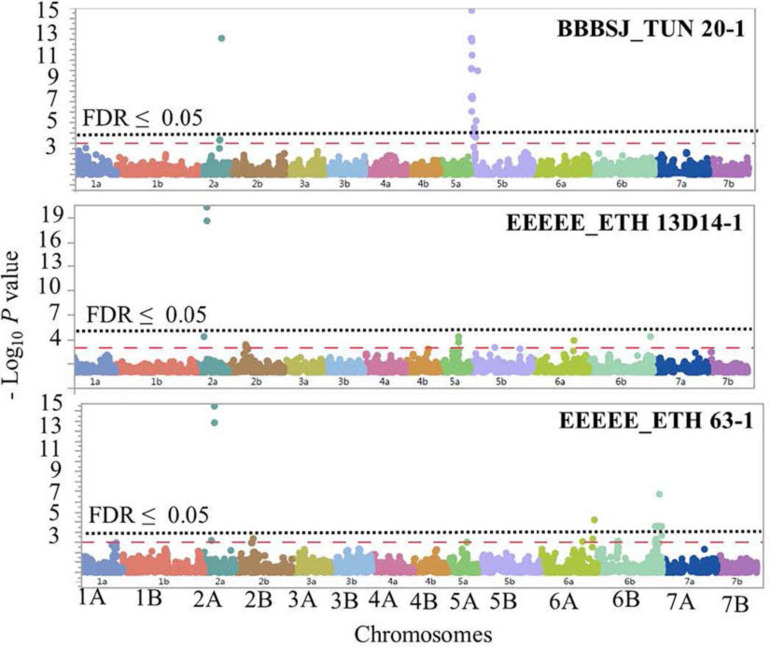
Manhattan plots showing *P*-values for single-nucleotide polymorphism (SNP) markers associated with response to leaf rust in durum wheat genotypes to the three durum wheat type isolates BBBSJ_TUN 20-1, EEEEE_ETH 13D14-1, and EEEEE_ETH 63-1. The horizontal dashed red line indicates significance level at *P*-value ≤ 0.001. The horizontal dotted black line indicates significance level at FDR ≤ 0.05.

The GWAS based on the linearized ITs to the three *Pt* isolates BBBSJ_TUN 20-1, EEEEE_ETH 13D14-1, and EEEEE_ETH 63-1 identified 64 significant SNPs (MTAs) at FDR ≤ 0.05. Based on the LD between significant markers, these MTAs represented six loci located on chromosome arms 2AS, 2AL, 5BS, 6AL, and 6BL. The most significant marker/locus explained 6–31% of phenotypic variation (Table 2, Figure 6, and Supplementary Table 6). Chromosome arms 5BS and 6BL carried most of the MTAs. Therefore, the pairwise LD between the significant markers on each of these chromosome arms were presented in Supplementary Figure 3 that was used to determine the number of loci on chromosomes 5BS and 6BL.

**TABLE 2:** Summary of leaf rust resistance loci in the durum wheat genotypes.

													**Svevo genome v1^i^**	
***P. triticina* race**	**Locus**	**Num. SNPs/locus^a^**	**Tag-SNP ^b^**	**Chr.^c^**	**SNP alleles^d^**	**SNP major allele**	**SNP minor allele**	**MAF^e^**	**Position (cM)^f^**	**–Log10 (*P-*value)**	***R*^2g^**	**pFDR^h^**	**Start**	**End**
BBBSJ_TUN 20-1	*QLrdu.2AL*	1	*IWB38096*	2A	T/C	T	C	0.16	197.6	13.1	0.22	1.5E-10	NA	NA
BBBSJ_TUN 20-1	*QLrdu.5BS-1*	26	*IWB47425*	5B	A/C	A	C	0.16	2.0	15.8	0.25	1.8E-12	5,077,659	5,077,759
BBBSJ_TUN 20-1	*QLrdu.5BS-2*	19	*IWB26157*	5B	A/G	G	A	0.06	16.7	5.2	0.08	2.3E-03	21,105,871	21,105,971
EEEEE_ETH 13D14-1	*QLrdu.2AS*	2	*IWB10489*	2A	T/C	C	T	0.08	67.5	20.3	0.31	5.0E-17	61,159,123	61,159,023
EEEEE_ETH 63-1	*QLrdu.2AS*	2	*IWB10489*	2A	T/C	C	T	0.08	67.5	16.5	0.26	3.6E-13	61,159,123	61,159,023
EEEEE_ETH 63-1	*QLrdu.6AL*	1	*IWB24755*	6A	T/C	C	T	0.03	129.4	5.2	0.09	1.7E-02	612,235,063	612,235,163
EEEEE_ETH 63-1	*QLrdu.6BL*	13	*IWB52926*	6B	A/G	G	T	0.04	154.6	7.8	0.13	6.4E-05	695,708,680	695,708,580

*^*a*^Details on all significant SNPs/locus were described in Supplementary Table 6. ^*b*^Significant single-nucleotide polymorphism (SNP) with smallest marker-trait association *P* values per locus. ^*c*^Chromosome arm of the locus. ^*d*^Alleles of the most significant marker in the loci. The underlined allele is the allele associated with resistance. ^*e*^Minor allele frequency of the most significant SNP/locus. ^*f*^SNP position based on the tetraploid consensus map of Maccaferri et al. (2015). ^*g*^The proportion of phenotypic variation explained by the most significant SNP in the locus. ^*h*^*P*-value of the false discovery rate of the most significant SNP in the locus. ^*i*^Physical position of SNP sequence based on the durum wheat genome sequence of Svevo available on International Wheat Genome Sequencing Consortium (Maccaferri et al., 2019).*

On chromosome arm 2AS, the large-effect loci, *QLrdu.2AS* (Tag SNP: *IWB10489*, 67.5 cM, 61 Mbp) was associated with response to the Ethiopian isolates EEEEE_ETH 13D14-1 and EEEEE_ETH 63-1. On chromosome arm 2AL, *QLrdu.2AL* (*IWB38096*, 197.6 cM) was associated with response to race BBBSJ_TUN 20-1. On chromosome arm 5BS, two loci were associated with response to BBBSJ_TUN 20-1 and designated as *QLrdu.5BS-1* (*IWB47425*) and *QLrdu.5BS-2* (*IWB26157*). *QLrdu.5BS-1* explained higher phenotypic variation compared to *QLrdu.5BS-2.* These two loci spanned a genomic region from 2.0 to 35.8 cM corresponding to 4–21 Mbp on Svevo physical map (Maccaferri et al., 2019). On chromosome arm 6AL, a small-effect locus, *QLrdu.6AL* (*IWB24755*, 129.4 cM, 612 Mbp) was associated with response to EEEEE_ETH 63-1. An additional locus on chromosome arm 6BL, *QLrdu.6BL* (*IWB52926*, 154.6 cM, 696 Mbp) was also associated with response to EEEEE_ETH 63-1. All the leaf rust resistance loci identified in this study were race/isolate specific, except *QLrdu.2AS* that was associated with two Ethiopian isolates (Table 2, Figure 6, and Supplementary Table 6).

The postulation of the six *Lr* loci in each genotype in this germplasm was based on the most significant marker per locus and is presented in Supplementary Table 1. We found that all genotypes carry at least one of the identified loci in this study except lines D06707, D06710, D091721, and D97780. A total of 91% of the genotypes carry *QLrdu.6AL* and *QLrdu.6BL*, whereas 88% of the genotypes carry *QLrdu.2AS*, *QLrdu.6AL*, and *QLrdu.6BL.* Nine genotypes carry all the six identified loci in this study including Plaza (PI 613619), D98015, D98016, D01279, D011238, D03004, D05547, D101558, and D101650.

#### Association Analysis for Stripe Rust Response

**FIGURE 7:**
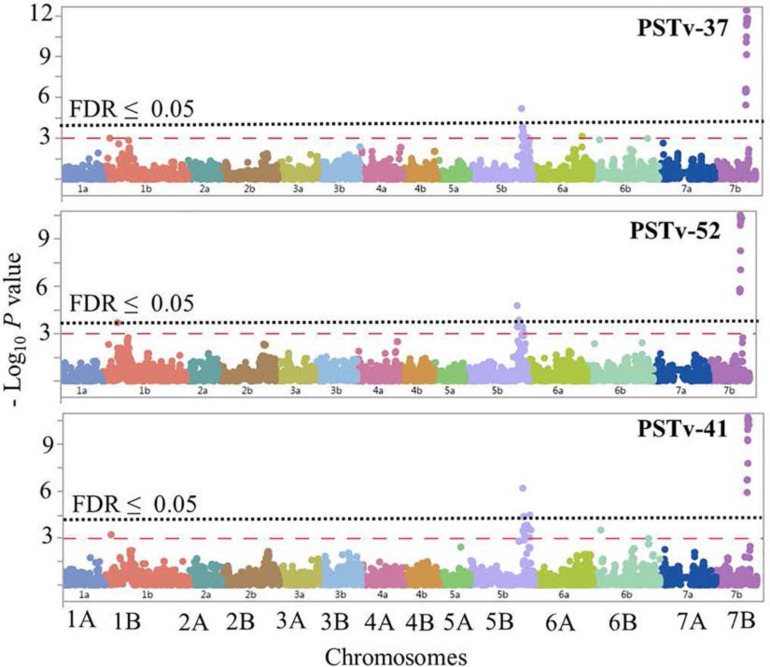
Manhattan plots showing *P*-values for single-nucleotide polymorphism (SNP) markers associated with response to stripe rust in durum wheat genotypes to the three *Pst* isolates PSTv-37, PSTv-52, and PSTv-41. The horizontal dashed red line indicates significance level at *P*-value ≤ 0.001. The horizontal dotted black line indicates significance level at FDR ≤ 0.05.

The GWAS to the three *Pst* isolates PSTv-37, PSTv-52, and PSTv-41 identified 46 significant MTAs, corresponding to four loci located on chromosome arms 1BS, 5BL, and 7BL. The most significant SNP/locus explained 6–19% of phenotypic variation (Table 3, Figure 7, and Supplementary Table 7). Most of the MTAs were on chromosome arms 5BL and 7BL. Therefore, the pairwise LD between the significant markers on each of these chromosome arms were presented in Supplementary Figure 4 that was used to determine the number of loci on each chromosome.

**TABLE 3:** Summary of stripe rust resistance loci in the durum wheat genotypes.

													**Svevo genome v1^i^**	
***Pst* race**	**Locus**	**Num. SNPs/locus^a^**	**Tag-SNP^b^**	**Chr.^c^**	**SNP alleles^d^**	**SNP major allele**	**SNP Minor allele**	**MAF^e^**	**Position (cM)^f^**	**–Log_10_ (*P-*value)**	***R*^2g^**	**pFDR^h^**	**Start**	**End**
PSTv-37	*QYrdu.5BL-1*	7	*IWA6271*	5B	A/G	G	A	0.2	187.1	5.19	0.08	1.92E-03	681,513,107	681,512,935
PSTv-37	*QYrdu.7BL*	33	*IWB10533*	7B	T/C	C	T	0.4	187.5	11.82	0.19	1.27E-09	696,924,344	696,924,444
PSTv-52	*QYrdu.1BS*	1	*IWB31649*	1B	A/G	A	G	0.2	33.0	3.70	0.06	5.02E-02	88,957,108	88,957,007
PSTv-52	*QYrdu.5BL-1*	9	*IWA6271*	5B	A/G	G	A	0.2	187.1	4.79	0.08	4.94E-03	681,513,107	681,512,935
PSTv-52	*QYrdu.7BL*	33	*IWB10533*	7B	T/C	C	T	0.4	187.5	10.33	0.17	2.91E-08	696,924,344	696,924,444
PSTv-41	*QYrdu.5BL-1*	9	*IWA6271*	5B	A/G	G	A	0.2	187.1	6.21	0.10	1.92E-04	681,513,107	681,512,935
PSTv-41	*QYrdu.5BL-2*	1	*IWB64287*	5B	A/C	C	A	0.1	193.4	4.49	0.07	9.30E-03	691,154,062	691,153,962
PSTv-41	*QYrdu.7BL*	33	*IWB10533*	7B	T/C	C	T	0.4	187.5	10.26	0.17	3.35E-08	696,924,344	696,924,444

*^*a*^Details on all significant SNPs/locus were described in Supplementary Table 7. ^*b*^Significant single-nucleotide polymorphism (SNP) with smallest marker-trait association *P-*values per locus. ^*c*^Chromosome arm of the locus. ^*d*^Alleles of the most significant marker in the locus. The underlined allele is the allele associated with resistance. ^*e*^Minor allele frequency of the most significant SNP/locus. ^*f*^SNP position based on the tetraploid consensus map of Maccaferri et al. (2015). ^*g*^The proportion of phenotypic variation explained by the most significant SNP in the locus. ^*h*^*P*-value of the false discovery rate of the most significant SNP in the locus. ^*i*^Physical position of SNP sequence based on the durum wheat genome sequence of Svevo available on International Wheat Genome Sequencing Consortium (Maccaferri et al., 2019).*

On chromosome arm 1BS, *QYrdu.1BS* (Tag SNP: *IWB31649*, 33 cM, 89 Mbp) was associated with response to race PSTv-52. On chromosome 5BL, two loci were detected. *QYrdu.5BL-1* (*IWA6271*, 187.1 cM, 682 Mbp) was associated with response to the three *Pst* races, whereas *QYrdu.5BL-2* (*IWB64287*, 193.4 cM, 691 Mbp) was associated with response to race PSTv-41. On chromosome 7BL, *QYrdu.7BL* (*IWB10533*, 187.5 cM, 697 Mbp) was associated with response to the three *Pst* races and explained most of the phenotypic variations. Two of the four identified stripe rust resistance loci in this study, *QYrdu.5BL-1* and *QYrdu.7BL* were associated with response to the three *Pst-*races, whereas the reaming *QYrdu.1BS* and *QYrdu.5BL-2* were race specific (Table 3, Figure 7, and Supplementary Table 7).

The postulation of the four *Yr* loci in each genotype in this germplasm was based on the most significant marker per locus and is presented in Supplementary Table 2. All genotypes carry at least one of the identified *Yr* loci in this study. A total of 78% of the genotypes carry *QYrdu.5BL-1* and *QYrdu.5BL-2*, whereas 52% of the genotypes carry *QYrdu.5BL-1* and *QYrdu.5BL-2* and *QYrdu.7BL*. Twenty-six genotypes carry all the four *Yr* loci identified in this study.

#### Association Analysis for Stem Rust Response

**FIGURE 8:**
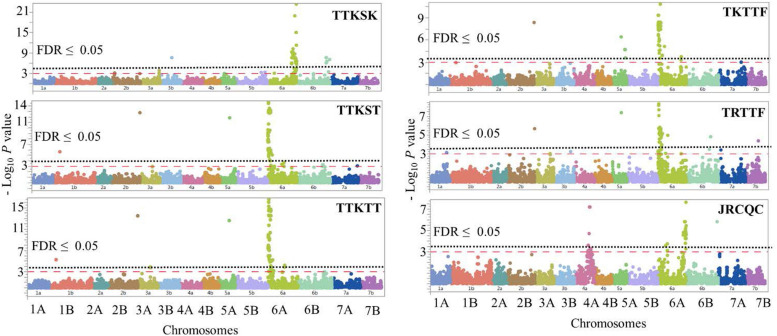
Manhattan plots showing *P*-values for single-nucleotide polymorphism (SNP) markers associated with response to stem rust in durum wheat genotypes to the three *Pgt* races TTKSK, TTKST, TTKTT, TKTTF, TRTTF, and JRCQC. The horizontal dashed red line indicates significance level at *P*-value ≤ 0.001. The horizontal dotted black line indicates significance level at FDR ≤ 0.05.

The GWAS detected 260 significant markers (MTAs), underlying 22 putative loci that were associated with stem rust response to the six *Pgt* races (TTKSK, TTKST, TTKTT, TKTTF, TRTTF, and JRCQC) (Table 4, Figure 8, and Supplementary Table 8). The highest number of MTAs were on chromosome arms 6AS (98 MTAs, three loci), 6AL (129 MTAs, three loci), 5AL (12 MTAs, three loci), and 6BL (seven MTAs, three loci). The pairwise LD between the significant markers on each of these chromosome arms were presented in Supplementary Figure 5 and were used to determine the number of loci per chromosome. Other MTAs were identified on chromosomes 3AL (three MTAs, two loci), 4AL (four MTAs, a single locus), 5BL (two MTAs, two loci), and 7BL (two MTAs, a single locus). Each of the chromosome arms 1BL, 2BL, and 3BL carried a single MTA. Of the 22 identified loci, seven loci, *QSrdu.2BL*, *QSrdu.4AL*, *QSrdu.5AL-1*, *QSrdu.6AS-1*, *QSrdu.6AL-2*, *QSrdu.6AL-3*, and *QSrdu.6BL-3*, were the most important loci in this study as they explained high phenotypic variations and/or associated with response to multiple *Pgt* races. These seven large-effect loci (highlighted in bold in Table 4) are the most robust *Sr* loci and were well represented in this germplasm (MAF ≥ 19%).

**TABLE 4:** Summary of stem rust resistance loci in the durum wheat genotypes.

													**Svevo genome v1^j^**	
***Pgt* race**	**Locus^a^**	**Num. SNPs/locus^b^**	**Tag-SNP^c^**	**Chr.^d^**	**SNP alleles^e^**	**SNP major allele**	**SNP minor allele**	**MAF^f^**	**Position (cM)^g^**	**–Log_10_ (*P*-value)**	***R*^2h^**	**pFDR^i^**	**Start**	**End**
TTKSK	*QSrdu.3AL-2*	1	*IWB72044*	3A	A/G	A	G	0.05	177.9	3.85	0.06	1.64E-02	736,648,215	736,64,8115
TTKSK	*QSrdu.3BL*	1	*IWB49397*	3B	T/G	T	G	0.05	77.1	7.61	0.12	4.73E-06	370,964,536	370,964,636
TTKSK	*QSrdu.5BL-1*	1	*IWB9652*	5B	T/C	C	T	0.08	181.5	3.28	0.05	5.42E-02	674,697,988	674,698,088
TTKSK	*QSrdu.5BL-2*	1	*IWB64287*	5B	A/C	C	A	0.07	193.4	3.36	0.05	4.58E-02	691,154,062	691,153,962
TTKSK	***QSrdu.6AL-2***	92	*IWB69393/dCAPS_Sr13*	6A	T/C	T	C	0.19	128.9	19.92	0.32	6.57E-17	611,710,729	611,710,829
TTKSK	*QSrdu.6BL-2*	6	*IWB5378*	6B	T/G	G	T	0.05	146.0	7.61	0.12	4.73E-06	682,240,129	682,240,229
TTKSK	*QSrdu.6BL-3*	1	*IWB46893*	6B	A/G	G	A	0.38	155.1	3.46	0.05	3.65E-02	693,337,728	693,337,628
TTKST	*QSrdu.1BL*	1	*IWB50554*	1B	A/G	G	A	0.11	27.6	5.66	0.09	3.28E-04	NA	NA
TTKST	***QSrdu.2BL***	1	*IWB48212*	2B	A/C	A	C	0.20	193.6	12.77	0.20	4.11E-11	789,417,490	789,417,417
TTKST	***QSrdu.5AL-1***	1	*IWB62132*	5A	T/G	G	T	0.21	136.3	11.84	0.18	3.25E-10	532,077,979	532,077,878
TTKST	***QSrdu.6AS-1***	80	*IWB10558/KASP_6AS_IWB10558*	6A	T/C	C	T	0.20	0.2	14.71	0.23	1.26E-11	1,590,026	1,590,126
TTKST	*QSrdu.6AS-2*	3	*IWB67075*	6A	A/G	A	G	0.09	34.9	3.41	0.05	4.99E-02	50,134,208	50,134,274
TTKST	*QSrdu.6AS-3*	1	*IWA7295*	6A	T/G	T	G	0.03	45.9	3.67	0.05	2.76E-02	86,025,214	86,025,359
TTKTT	*QSrdu.1BL*	1	*IWB50554*	1B	A/G	G	A	0.11	27.6	5.28	0.08	7.78E-04	NA	NA
TTKTT	***QSrdu.2BL***	1	*IWB48212*	2B	A/C	A	C	0.20	193.6	13.49	0.21	7.67E-12	789,417,490	789,417,417
TTKTT	*QSrdu.3AL-1*	2	*IWB36155*	3A	T/C	T	C	0.04	90.4	3.91	0.06	1.45E-02	572,456,904	572,456,785
TTKTT	***QSrdu.5AL-1***	1	*IWB62132*	5A	T/G	G	T	0.21	136.3	12.61	0.20	5.60E-11	532,077,979	532,077,878
TTKTT	***QSrdu.6AS-1***	80	*IWA5416/KASP_6AS_ IWB10558*	6A	T/C	T	C	0.21	0.2	16.62	0.26	2.62E-13	1,198,024	1,197,947
TTKTT	*QSrdu.6AS-3*	10	*IWA7295*	6A	T/G	T	G	0.03	45.9	4.21	0.06	8.39E-03	86,025,214	86,025,359
TKTTF	***QSrdu.2BL***	1	*IWB48212*	2B	A/C	A	C	0.20	193.6	8.36	0.13	1.40E-06	789,417,490	789,417,417
TKTTF	***QSrdu.5AL-1***	1	*IWB62132*	5A	T/G	G	T	0.21	136.3	6.42	0.10	6.58E-05	532,077,979	532,077,878
TKTTF	*QSrdu.5AL-2*	10	*IWB2075*	5A	A/G	A	G	0.03	183.0	4.72	0.07	2.61E-03	623,114,829	623,114,760
TKTTF	*QSrdu.5AL-3*	1	*IWB14445*	5A	T/G	G	T	0.04	197.7	3.60	0.05	3.04E-02	640,125,144	640,125,045
TKTTF	***QSrdu.6AS-1***	74	*IWB60233/KASP_6AS_ IWB10558*	6A	T/C	T	C	0.12	0.9	10.83	0.19	1.60E-07	3,721,352	3,721,450
TKTTF	*QSrdu.6AL-1*	3	*IWB31531*	6A	A/G	A	G	0.08	122.1	3.75	0.06	2.21E-02	600,285,732	600,285,802
TRTTF	***QSrdu.2BL***	1	*IWB48212*	2B	A/C	A	C	0.20	193.6	5.69	0.09	5.84E-04	789,417,490	789,417,417
TRTTF	***QSrdu.5AL-1***	1	*IWB62132*	5A	T/G	G	T	0.21	136.3	7.40	0.12	1.09E-04	532,077,979	532,077,878
TRTTF	***QSrdu.6AS-1***	58	*IWB53754/KASP_6AS_ IWB10558*	6A	A/G	G	A	0.21	0.2	8.34	0.13	3.58E-05	1,202,823	1,202,923
TRTTF	*QSrdu.6BL-1*	5	*IWB21973*	6B	A/G	A	G	0.16	103.7	4.84	0.08	2.78E-03	621,527,086	621,527,186
TRTTF	*QSrdu.7BL*	2	*IWB17567*	7B	T/G	G	T	0.05	147.0	4.40	0.07	7.19E-03	675,357,404	675,357,554
JRCQC	***QSrdu.4AL***	4	*IWA4651*	4A	A/G	A	G	0.33	162.4	7.20	0.11	1.70E-04	718,619,698	718,619,565
JRCQC	***QSrdu.6AL-3***	39	*IWB41394/dCAPS_Sr13_ R2nocut/dCAPS_Sr13_R1cut*	6A	T/C	T	C	0.38	129.4	5.83	0.09	1.16E-03	613,212,821	613,212,744
JRCQC	***QSrdu.6BL-3***	1	*IWB46893*	6B	A/G	G	A	0.38	155.1	5.83	0.09	1.16E-03	693,337,728	693,337,628

*^*a*^The loci in bold represent the most important stem rust resistance loci identified in this study. ^*b*^Details on all significant SNPs/locus were described in Supplementary Table 7. ^*c*^Significant single-nucleotide polymorphism (SNP) with smallest marker-trait association *P* values per locus. ^*d*^Chromosome arm of the locus. ^*e*^Alleles of the most significant marker in the locus. The underlined allele is the allele associated with resistance. ^*f*^Minor allele frequency of the most significant SNP/locus. ^*g*^SNP position based on the tetraploid consensus map of Maccaferri et al. (2015). ^*h*^The proportion of phenotypic variation explained by the most significant SNP in the locus. ^*i*^*P*-value of the false discovery rate of the most significant SNP in the locus. ^*j*^Physical position of SNP sequence based on the durum wheat genome sequence of Svevo available on International Wheat Genome Sequencing Consortium (Maccaferri et al., 2019).*

The most important large-effect locus identified on the distal end of chromosome arm 6AS was *QSrdu.6AS-1* (58–80 MTAs, Tag-SNP: *IWB10558*, 0.2 cM, 2 Mbp) that was associated with response to races TTKST, TTKTT, TKTTF, and TRTTF but not to race TTKSK and JRCQC. *KASP_6AS_IWB10558* linked to the gene *Sr8155B1* was among the most significant markers in this locus. In addition, *KASP_6AS_IWB10558* was in high LD with other significant markers in *QSrdu.6AS-1* (Table 4, Supplementary Table 8, and Supplementary Figure 5) suggesting that the latter locus is indeed *Sr8155B1*. Two additional small-effect loci on chromosome 6AS and proximal to *QSrdu.6AS-1* were identified. *QSrdu.6AS-2* (*IWB67075*, 34.9 cM, 50 Mbp) was associated with response to race TTKST, whereas *QSrdu.6AS-3* (*IWA7295*, 45.9 cM, 86 Mbp) was associated with response to both races TTKST and TTKTT (Table 4, Figure 8, and Supplementary Table 8).

Two large-effect loci appeared on chromosome arm 6AL. *QSrdu.6AL-2* (Tag-SNP: *IWB69393*, 128.9 cM, 612 Mbp) was associated with response to race TTKSK, while *QSrdu.6AL-3* (Tag-SNP: *IWB41394*, 129.4 cM, 613 Mbp) was associated with response to race JRCQC. An additional small-effect locus on chromosome 6AL, *QSrdu.6AL-1* (Tag-SNP: *IWB31531*, 122.1 cM, 600 Mbp) was associated with response to race TKTTF. Even though, *QSrdu.6AL-2* and *QSrdu.6AL-3* were close based on their genetic positions (on tetraploid consensus map) and physical positions (Svevo genome v1), significant markers in these two loci were not in strong LD (*R*^2^ = 0.14, Supplementary Figure 5). *QSrdu.6AL-2* and *QSrdu.6AL-3* appeared to be associated with *Sr13* gene/alleles. This is because *Sr13* diagnostic marker (*dCAPS_Sr13*) was among the most significant markers for race TTKSK and in LD with other significant SNPs in *QSrdu.6AL-2*. *Sr13* allele markers, *dCAPS_Sr13_R1cut* (identifying *Sr13a-*R1 allele) and *dCAPS_Sr13_R2nocut* (identifying *Sr13* R2 allele or *Sr13b*) were among significant markers for race JRCQC and in LD with significant SNPs in *QSrdu.6AL-3* (Table 4, Figure 8, and Supplementary Table 8).

The major allele (‘T’) of *IWB41394* that is the most significant SNP in *QSrdu.6AL-3* and present in 62 % of the durum genotypes was associated with susceptibility to race JRCQC. On the other hand, the most significant marker in *QSrdu.6AL-3* was *Sr13b* marker *dCAPS_Sr13_R2nocut.* The latter showed that 58% of the durum lines carry *Sr13b* associated with susceptibility to race JRCQC. Therefore, it is likely that the ‘T’ allele of *IWB41394* is associated with *Sr13b.* Overall, in 90.3% of the genotypes, there was agreement between marker *dCAPS_Sr13_R2nocut* and marker *IWB41394* in postulating *Sr13b* allele (Supplementary Table 9).

On chromosome arm 4AL, *QSrdu.4AL* (Tag-SNP: *IWA4651*, 162.4 cM, 719 Mbp) was another large-effect locus identified for response to race JRCQC. On chromosome arm 5AL, three loci were identified. *QSrdu.5AL-1* (*IWB62132*, 136.3 cM, 532 Mbp) was associated with response to multiple races TTKST, TTKTT, TRTTF, and TKTTF and explained 10–20% of phenotypic variation. In addition, two small-effect loci on chromosome 5AL, *QSrdu.5AL-2* (*IWB2075*, 183.0 cM, 623 Mbp) and *QSrdu.5AL-3* (*IWB14445*, 197.7 cM, 640 Mbp) were associated with response to race TKTTF. On chromosome arm 6BL, *QSrdu.6BL-1* (*IWB21973*, 103.7 cM, 622 Mbp) and *QSrdu.6BL-2* (*IWB5378*, 146.0 cM, 682 Mbp) was associated with response to race TRTTF and TTKSK, respectively. *QSrdu.6BL-3* (*IWB46893*, 155.1 cM, 693 Mbp) was associated with responses to races TTKSK and JRCQC. The major allele of the most significant marker in *QSrdu.6BL-3*, *IWB46893*, was associated with resistance to TTKSK but with susceptibility to JRCQC.

On chromosome arm 5BL, two small-effect loci were identified: *QSrdu.5BL-1* (*IWB9652*, 181.5 cM, 675 Mbp) and *QSrdu.5BL-2* (*IWB64287*, 193.4 cM, 691 Mbp). Interestingly, *IWB64287* was also associated with response to *Pst*-race PSTv-41 (Tables 3, 4). This suggests that this locus on 5BL at 691 Mbp is associated with response to both stripe rust and stem rust and the allele ‘C’ of marker *IWB64287* provides resistance to both rust pathogens. Few MTAs were identified on each of the chromosomes 1BL, 2BL, 3AL, 3BL, and 7BL and most of these associations had minor effects on disease response (6–12%), except *QSrdu.2BL* (*IWB48212*, 193.6 cM, 789 Mbp) that explained relatively higher phenotypic variations (9–21%) to races TTKST, TTKTT, TKTTF, and TRTTF. Of the 22 identified loci for stem rust, five (*QSrdu.1BL*, *QSrdu.2BL*, *QSrdu.5AL-1*, *QSrdu.6AS-1*, and *QSrdu.6BL-3*) were associated with response to more than one race while the remaining loci were race specific (Table 4, Figure 8, and Supplementary Table 8).

#### Frequencies of Sr8155B1, Sr13, and Sr7b in the Durum Wheat Genotypes and Their Marker Accuracies

Gene postulation for *Sr8155B1*, *Sr13* alleles, and *QSrdu.4AL* in each of the durum wheat genotypes is presented in Supplementary Tables 3, 5. Both phenotypic data (Supplementary Table 3) and marker data (Supplementary Table 9) were used to postulate the gene combinations present in each of the durum wheat genotypes. For the genotypic data, the markers *dCAPS_Sr13*, *dCAPS _Sr13_R1cut, dCAPS_Sr13_R2nocut*, *dCAPS_Sr13_R3nocut*, *KASP_6AS_IWB10558*, and *IWA4651* were used to postulate *Sr13a-R1*, *Sr13b*, *Sr13a-R3*, *Sr8155B1*, and *QSrdu.4AL* (designated in this study as *Sr7b*), respectively. We found that 81, 79, and 64% of the durum wheat genotypes carry *Sr13*, *Sr8155B1*, and *Sr7b*, respectively. A single breeding line (D07726) does not carry any of these genes.

A total of 61% of the durum genotypes carry an *Sr13* allele and *Sr8155B1*, whereas 50% of the durum collection carry an *Sr13* allele and *Sr7b.* We found that 54% of the durum genotypes have a least *Sr8155B1* and *Sr7b* and 40% of the genotypes have the three genes *Sr13*, *Sr8155B1*, and *Sr7b.* Based on *Sr13* allele markers, *Sr13* functional alleles *Sr13a-R1*, *Sr13b*, and *Sr13a-R3* were identified in the durum genotypes. *Sr13b* was the most common allele, being present in 56% of the durum genotypes. *Sr13a-R1* and *Sr13a-R3* were less frequent and occurred in only 17 and 7% of the durum accessions, respectively (Supplementary Table 9).

Because gene postulation for these three genes was possible based only on the phenotype, we determined the accuracies of markers *dCAPS_Sr13*, *IWB69393*, *KASP_6AS_IWB10558*, and *IWA4651*. For the gene *Sr13*, the accuracy for *dCAPS_Sr13* and *IWB69393* was 100 and 95% (3% false positives and 2% false negatives), respectively. For *Sr8155B*1, the marker *KASP_6AS_IWB10558* had an accuracy of 99.6% (0.4% false positives), whereas for *Sr7b*, the marker *IWA4651* had an accuracy of 98.8% (1.2% false positives). The postulation of the remaining three large-effect *Sr* loci in each genotype (Supplementary Table 3) showed that 30 genotypes carry *Sr8155B1*, *Sr13*, *Sr7b*, *QSrdu.2BL*, *QSrdu.5AL-1*, and *QSrdu.6BL-3*.

## Discussion

### Leaf Rust Resistance in Durum Wheat Genotypes

All the durum genotypes were resistant to the common wheat type race MBDSS that is widely distributed in the wheat growing regions of the United States (Kolmer and Hughes, 2014). This agrees with previous studies indicating that *Pt*-isolates from common wheat are generally avirulent on durum wheat (Singh, 1991; Huerta-Espino and Roelfs, 1992; Ordoñez and Kolmer, 2007a; Aoun et al., 2016). Herrera-Foessel et al. (2014) reported that most of the CIMMYT durum wheat germplasm carry *Lr72* that is effective against common wheat type races. Thus, *Lr72* could be also present in the durum wheat genotypes in this study. Many of the genotypes in our study were susceptible to Mexican, Moroccan, Tunisian, and Ethiopian durum wheat type isolates. None of the durum genotypes were resistant to the *Pt-*Mexican race BBBQJ. The latter is similar to a race collected on durum wheat in California (Kolmer, 2013) and on hard red winter wheat in Kansas (Kolmer, 2015b). Even though *Pt-*race BBBQJ is not yet present in North Dakota, introgression of leaf rust resistance to this race in the NDSU durum wheat lines will help the growers in tackling in future challenges. For instance, previously identified *Lr* genes like those identified in CIMMYT germplasm (Herrera-Foessel et al., 2007, 2008a,b; Huerta-Espino et al., 2009) and in the USDA–National Small Grains Collection (NSGC) of durum wheat (Aoun et al., 2016, 2017, 2019) could be used to enhance leaf rust resistance to race BBBQJ in the NDSU durum wheat germplasm. The Ethiopian isolates of race EEEEE were virulent to only 10–28% of the durum genotypes. Even though, the two Ethiopian isolates in this study carry the same race (EEEEE) on Thatcher wheat differentials, there were differences in their virulence profiles on durum wheat genotypes in our study. These results agree with Aoun et al. (2020) observations showing that different virulence phenotypes were found within a collection of isolates of race EEEEE based on a set of durum wheat differentials.

Comparative mapping between the identified six all-stage leaf rust resistance loci in this study and designated wheat *Lr* genes showed that any of the two loci on chromosome 5BS could be *Lr52* that was previously identified in the durum wheat cultivar Wallaroi (Singh et al., 2010). Similarly, *QLrdu.6AL* is most likely *Lr64* that originated from wild emmer wheat (*Triticum dicoccoides*) (Dyck, 1994; McIntosh et al., 2009; Kolmer et al., 2019). The remaining loci did not map close to known *Lr* genes and thus could be novel. Comparison of the map locations suggests that *QLrdu.2AS* (67.5 cM, 61 Mbp) is likely the same locus which was earlier found associated with leaf rust response in durum wheat and tagged by the SSR marker *wmc522* (63.6 cM, 58 Mbp) (Maccaferri et al., 2010). The nine genotypes that carry all the six identified *Lr* loci in this study are useful to keep these resistance sources in future released varieties.

### Stripe Rust Resistance in Durum Wheat Genotypes

Many of the durum wheat genotypes (67–69%) in this study were resistant to the three U.S. *Pst* races (PSTv-37, PSTv-52, and PSTv-41). A previous study that screened a worldwide collection of elite durum wheat lines to six US and Italian *Pst-*races (including PSTv-37) showed that only 7.8–31.5% of the genotypes were resistant (Liu et al., 2017). This suggests that the durum wheat collection in this study had undergo selection to accumulate potentially useful loci for stripe rust resistance to the North American *Pst* races. The durum wheat responses to these three *Pst*- races used in this study were highly correlated, showing that the NDSU durum genotypes had a broad spectrum of stripe rust resistance.

With rapid and dangerous shifts in *Pst* populations globally (Solh et al., 2012), our study will help durum wheat breeding programs by providing new stripe rust resistance sources. We identified four loci associated with all-stage stripe rust resistance that did not correspond to any designated stripe rust resistance genes. At the same time, some of the loci identified in this study were mapped close to not yet characterized stripe rust resistance quantitative trait loci (QTL) in the literature. For instance, *QYrdu.1BS* (*IWB31649*, 33.0 cM) was located close to previously identified locus *Yrdurum-1BS.1* (34.1–40.1 cM) that was associated with stripe rust response in a worldwide collection of elite durum wheat (Liu et al., 2017). Similarly, the *QYrdu.5BL-1* (*IWA6271*, 187.1 cM, 682 Mbp) was mapped close to the stripe rust resistance QTL, *QYr.usw-5B* (*IWA7066*, 179.6 cM, 674 Mbp, Lin et al., 2018) that was earlier detected in the durum wheat line W9262-260D3 (Kyle^∗^2/Biodur). The position of *QYrdu.7BL* (*IWB10533*, 187.5 cM, 697 Mbp) also overlaps with that of *Yrdurum-7BL* (184.5–190.5 cM) that was associated with stripe rust response at seedling stage in elite durum wheat genotypes (Liu et al., 2017). At the similar location, Lin et al. (2018) also identified *QYr.usw-7B* (181.1 cM, 694 Mbp) in the durum wheat line W9262-260D3 (Kyle^∗^2/Biodur) to Canadian isolates at seedling stage and to Mexican races at adult-plant stage. Further research warrants to characterize the four stripe rust resistance loci detected in this study and study their relationship with those previously identified in the literature. The 26 genotypes that carry all the four *Yr* loci identified in this study are excellent sources to introgress these stripe rust resistance sources in future durum wheat varieties.

### Stem Rust Resistance in Durum Wheat Genotypes

The majority of durum wheat genotypes were resistant to the three Ug99-lineage races TTKSK, TTKST, and TTKTT. Interestingly, 19% of the genotypes were susceptible to race TTKSK while only 1% of the genotypes were susceptible to the other two Ug-99 lineage races TTKST and TTKTT. This suggests that these durum advanced breeding lines carry stem rust resistance gene(s)/allele(s), such as *Sr8155B1*, that are effective against TTKST and TTKTT but ineffective against TTKSK. Therefore, a combination of multiple *Sr* genes in the newly developed durum wheat cultivars is recommended for effective resistance to different races of the Ug99 lineage. Similarly, only one line was susceptible to the Digalu race (TKTTF) (Olivera et al., 2015). The durum genotypes were all resistant to race TRTTF. In contrast to TRTTF, race JRCQC that is adapted to durum wheat (Hundie et al., 2019) in Ethiopia was the most virulent race on the durum genotypes in our study. This suggests that *Sr* genes/alleles effective to races TTKSK, TTKST, TTKTT, TKTTF, and TRTTF do not provide resistance to JRCQC. Olivera et al. (2015) showed that races JRCQC, TRTTF, and TKTTF are phylogenetically different from Ug99-lineage races. Therefore, *Sr* genes effective to each of these race lineages could be different. This implies that a combination of diverse *Sr* genes should be implemented in newly released cultivars.

The durum wheat genotypes in this study showed higher levels of stem rust resistance compared to germplasm collections used in previous studies. For example, in a durum wheat collection from different durum wheat-growing regions in Mediterranean countries, the Southwestern United States, and Mexico, 42.1, 18.6, and 52.5% of the tested accessions were susceptible to TTKSK, TRTTF, and JRCQC, respectively (Letta et al., 2014). In another study (Chao et al., 2017), most of the USDA– NSGC of durum wheat collection comprised of landraces, breeding lines, and cultivars were found susceptible to TTKSK (81.6%), TRTTF (72.1%), and JRCQC (90.6%). This shows that the NDSU breeding program selected for stem rust resistance to most of the *Pgt*-races used in this study. It was reported that resistance to the Ug99 lineage in the North American durum cultivars is mainly due to *Sr13* alleles that were first identified in durum wheat and was then transferred to hexaploid wheat (Knott, 1990). However, in our study we observed variations in the ITs to the *Pgt*-races. For instance, the most common resistant IT to races TTKST, TTKTT, TKTTF, TRTTF (IT = 0), indicative of *Sr8155B1*, was much lower compared to the most common resistant infection type to TTKSK (IT = 2–) and JRCQC (IT = 22^+^). This suggests that stem rust genetic architecture in this durum wheat collection is much more complex and multiple genes/alleles could be identified in this durum germplasm. In this germplasm, we found that 40% of the durum genotypes carry *Sr13*, *Sr8155B1*, and *Sr7b* and 30 genotypes (12%) carry large-effect loci identified in this study including *Sr8155B1*, *Sr13a*/*Sr13b*, *Sr7b*, *QSrdu.2BL*, *QSrdu.5AL-1*, and *QSrdu.6BL-3.* This gene/loci combination is critical to keep in future released durum wheat varieties. The remaining 15 *Sr* loci that explained low phenotypic variation or associated with relatively low MAF need to be first validated before being used in breeding programs.

Comparative mapping showed that out of the 22 identified all-stage stem rust resistance loci in this study, four loci corresponded to cataloged *Sr* genes/alleles. In addition, eight loci in this study were mapped close to previously detected stem rust resistance QTL that were not yet cataloged in wheat. *QSrdu.1BL* was also found close to the DArT marker *wPt-1876* (26.3 cM) that was associated with stem rust response in durum wheat (Letta et al., 2014). The locus *QSrdu.2BL* (*IWB48212*, 193.6 cM, 789 Mbp) was mapped close to SSR marker *wmc356* (788 Mbp) that has been found associated with stem rust response in durum wheat (Letta et al., 2014). Within the genomic regions of *QSrdu.3AL-1* (*IWB36155*, 90.4 cM, 572 Mbp) and *QSrdu.3AL-2 (IWB72044*, 177.9 cM, 737 Mbp), Letta et al. (2013) identified two stem rust resistance loci in durum wheat tagged with the SSR marker *wmc428* (93.8 cM, 589 Mbp) and DArt marker (*wPt-8203*, 178.3 cM). The locus *QSrdu.4AL* (*IWA4651*, 162.4 cM, 719 Mbp) that was associated with response to race JRCQC was close to the mapping position of *Sr7* locus (McIntosh et al., 1995; Saini et al., 2018) and it is likely *Sr7b*. We found that 64% of the durum genotypes carry *Sr7b* and it is important to keep it in future released varieties, especially that only few known genes confer resistance to race JRCQC. Within the genomic region of *QSrdu.4AL* (*Sr7b*), Letta et al. (2014) identified a locus tagged with the SSR marker *barc78* (161.7 cM, 656 Mbp) associated with response to race JRCQC at seedling stage in elite durum wheat panel. In the same durum wheat panel, Letta et al. (2013) identified two MTAs on chromosome arm 4AL tagged by the DArT markers *wPt-9196* (157.7 cM) and *wPt-0798* (161.7 cM) associated with stem rust response at adult-plant stage in field trials in Ethiopia. Proximal to the genomic region of *QSrdu.5AL-1*, a MTA represented with the SSR marker *gwm1570* (134.5 cM) was associated with stem rust seedling response in durum wheat (Letta et al., 2014). Similarly, the genomic region near *QSrdu.5AL-2* and *QSrdu.5AL-3* were found to carry two stem rust resistance loci tagged with markers *gwm126* (191.2 cM) and *gwm291* (205.0 cM) in durum wheat in field trials in Ethiopia (Letta et al., 2013). On chromosome 5BL and at a close genomic region to *QSrdu.5BL-2*, Letta et al. (2014) detected a GWAS hit tagged by DArt marker *wPt-0566* (191.6 cM) associated with stem rust seedling response in durum wheat (Letta et al., 2014).

The locus *QSrdu.6AS-1* (*KASP_6AS_IWB10558*, 0.2 cM, 2 Mbp) that was associated with resistance to race TTKST, TTKTT, TKTTF, and TRTTF was identified in the region of *Sr8155B1*. This gene was first identified in the durum wheat line 8155-B1 and known to confer resistance against race TTKST (Nirmala et al., 2017). The gene *Sr8155B1* was later reported in the durum wheat cultivar ‘Lebsock’ and provided resistance to race TRTTF (Saini et al., 2018). In our study, we observed that *Sr8155B1* provides resistance to additional *Pgt*-races TTKTT and TKTTF. In agreement with Nirmala et al. (2017), we found that this gene is common in the Midwestern durum wheat with 79% of the breeding lines and cultivars carrying this gene. Based on *Sr13* diagnostic markers, *QSrdu.6AL-2* and *QSrdu.6AL-3* were found to be associated with *Sr13* gene/alleles. *Sr13* is known to be common in North American and CIMMYT durum wheat cultivars (Jin, 2005; Singh et al., 2015) and is present in 84% of this durum wheat germplasm. *Sr13a* that confers resistance to JRCQC is present in only 17% of the durum genotypes in this study. However, 66% of the genotypes were resistant to JRCQC. This is most likely explained by the presence of other genes conferring resistance to JRCQC, e.g., *Sr7b*. *Sr13* gene/allele CAPS markers used in this study are difficult to be used in high-throughput genotyping for marker assisted selection. Therefore, the most significant SNPs in *QSrdu.6AL-2* (e.g., *IWB69393*) and *QSrdu.6AL-3* (e.g., *IWB41394*) can be converted into KASP or thermal asymmetric reverse PCR (STARP) markers to postulate the presence of *Sr13* gene and *Sr13b* allele, respectively.

The locus *QSrdu.7BL* (*IWB17567*, 147.0 cM, 675 Mbp) that was associated with response to race TRTTF is mapped close to the gene *Sr17*. The gene *Sr17* has been reported in tetraploid wheat and synthetic bread wheat (Bansal et al., 2008). However, race TRTTF is virulent to *Sr17*, therefore *QSrdu.7BL* is likely linked to *Sr17* or a new allele of *Sr17*. Close to the genomic region of *QSrdu.7BL*, Letta et al. (2013) also reported a stem rust resistance locus in durum wheat tagged by DArt marker *wPt-8615* (154.0 cM).

## Conclusion

We investigated the levels of all-stage resistance in durum wheat genotypes adapted to the Midwest region of the U.S. against six *Pt*-races, three *Pst*-races, and six *Pgt-*races. Many of the durum wheat breeding lines and cultivars were susceptible to durum wheat type *Pt* isolates, whereas all lines were resistant to the common wheat type *Pt* isolate. In contrast to leaf rust, many of the durum wheat genotypes has high levels of resistance to most stripe rust and stem rust pathogen races. Association mapping revealed six leaf rust resistance loci located on chromosomes 2AS, 2AL, 5BS, 6AL, and 6BL. Two of the loci are likely *Lr52* and *Lr64*, while the remaining four loci are most likely novel. Except *QLrdu.2AS*, the identified leaf rust resistance loci were race specific. For stripe rust, four loci were detected on chromosome arms 1BS, 5BL, and 7BL. All of these loci did not correspond to cataloged *Yr* genes. The loci *QYrdu.5BL-1* and *QYrdu.7BL* were associated with response to the three U.S. *Pst*-races used in this study. For stem rust, 22 resistance loci were detected on chromosomes 1BL, 2BL, 3AL, 3BL, 4AL, 5AL, 5BL, 6AS, 6AL, 6BL, and 7BL. Seven of these *Sr* loci had large effect and high frequencies in this germplasm, thus important to keep in future released durum wheat varieties. Our results showed the presence of known *Sr* genes *Sr8155B1*, *Sr13*, and *Sr7b* that were found together in 40% of this durum wheat germplasm. Seventeen *Sr* loci from this study are not yet cataloged and need to be validated and further characterized. Five of the identified stem rust resistance loci (*QSrdu.1BL*, *QSrdu.2BL*, *QSrdu.5AL-1*, *QSrdu.6AS-1*, and *QSrdu.6BL-3*) were associated with response to more than one race. The novel resistance loci identified in this study will enhance breeding for rust resistance in durum wheat. Because it is relatively easy to make crosses between tetraploid wheat and hexaploid wheat, new rust resistance genes identified in this durum wheat germplasm could also be transferred to common wheat. The SNP markers associated with the large-effect all-stage rust resistance genes/loci in this study can be converted to KASP or STARP markers for use in marker assisted breeding. The presence of gene pyramiding that is already present in this germplasm would be very valuable for breeding for rust resistance.

## Data Availability Statement

The datasets presented in this study can be found in online repositories. The names of the repository/repositories and accession number(s) can be found in the article/Supplementary Material.

## Author Contributions

MA and EE conceived and designed the experiments. MA, MR, and JK conducted the experiments. MA and AK analyzed the data. EE provided the resources. MA wrote the manuscript. All authors revised the manuscript.

## Conflict of Interest

The authors declare that the research was conducted in the absence of any commercial or financial relationships that could be construed as a potential conflict of interest.

## Footnotes

1 http://striperust.wsu.edu
