# Supplementary material for: Genome-Wide Association Studies Reveal All-Stage Rust Resistance Loci in Elite Durum Wheat Genotypes
Source: Front Plant Sci. 2021 Apr 12;12:640739. doi: 10.3389/fpls.2021.640739 (PMC8072158; doi:10.3389/fpls.2021.640739)
Supplement: Supplementary file 2 [file Data_Sheet_2.docx]

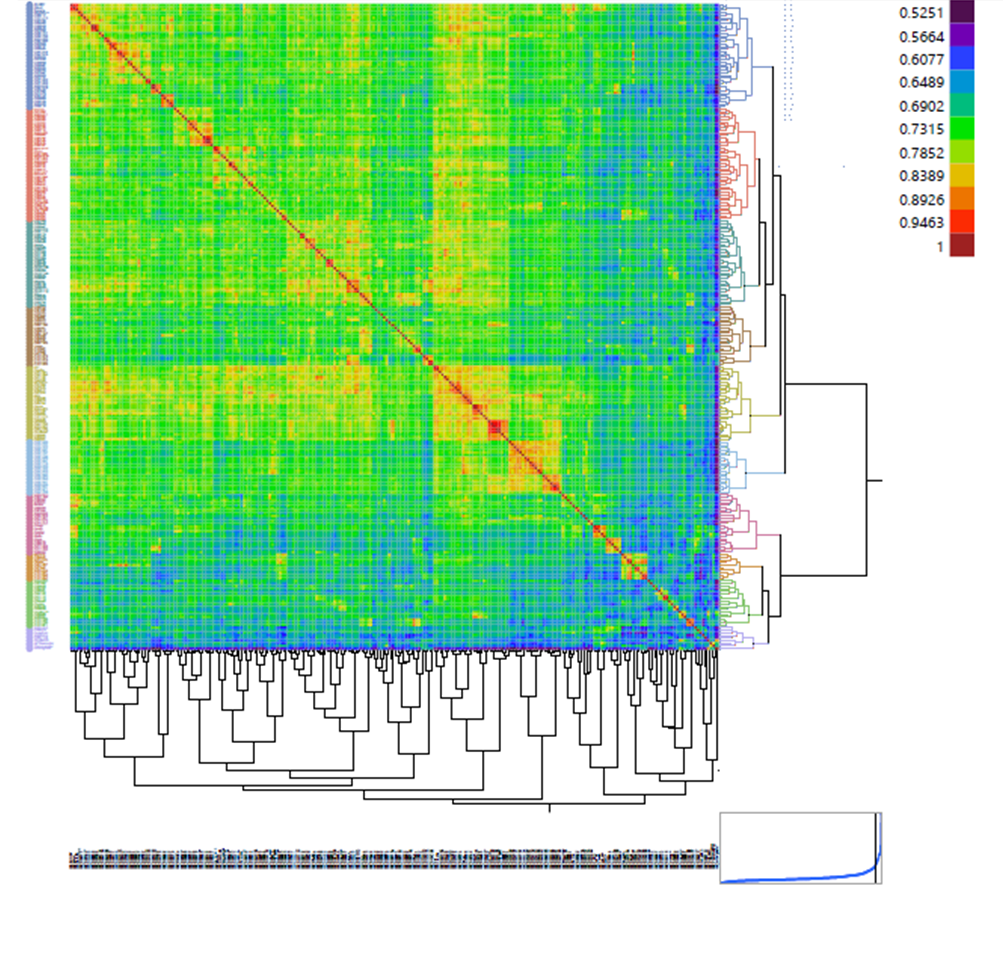


**SUPPLEMENTARY FIGURE S1** Kinship matrix among the 248 durum wheat genotypes displaying the relationship measures (identity-by-state) between each genotype pair.

**
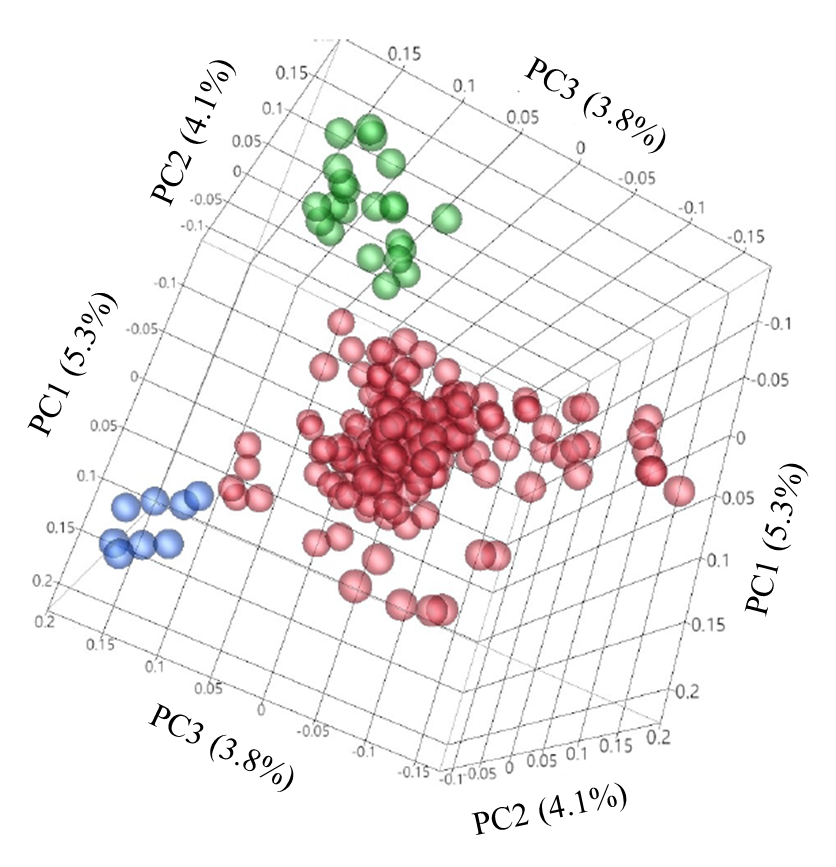
**

**SUPPLEMENTARY FIGURE S2** Principal component (PC) analysis plot illustrating the population structure in 248 durum wheat genotypes. The colors represent three different durum genotype groups. The PCA plot is constructed using 1, 457 SNPs (R^2^ ≤ 0.2). PC1, PC2, and PC3 explained 5.3%, 4.1%, and 3.8% of variation, respectively.


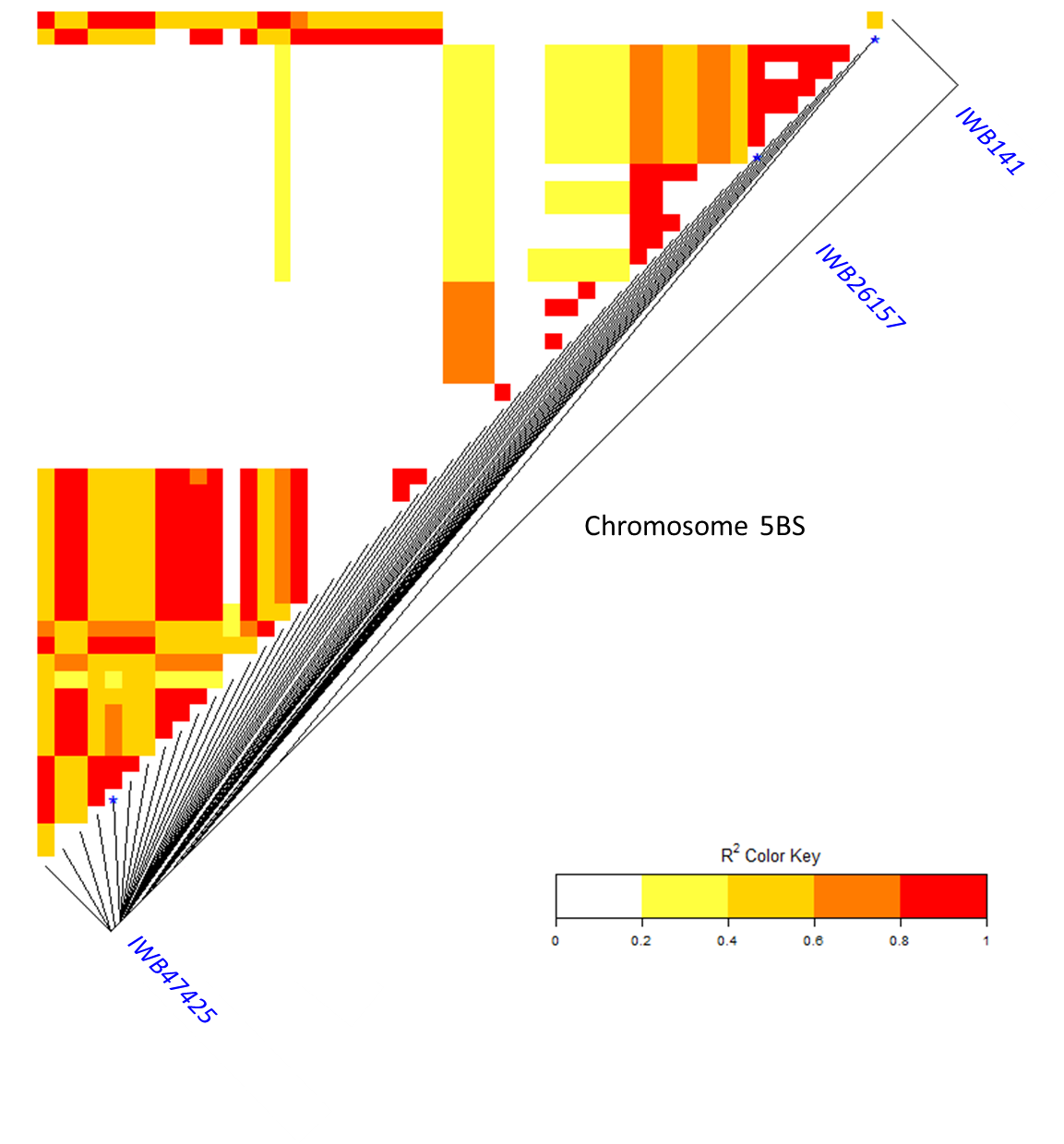


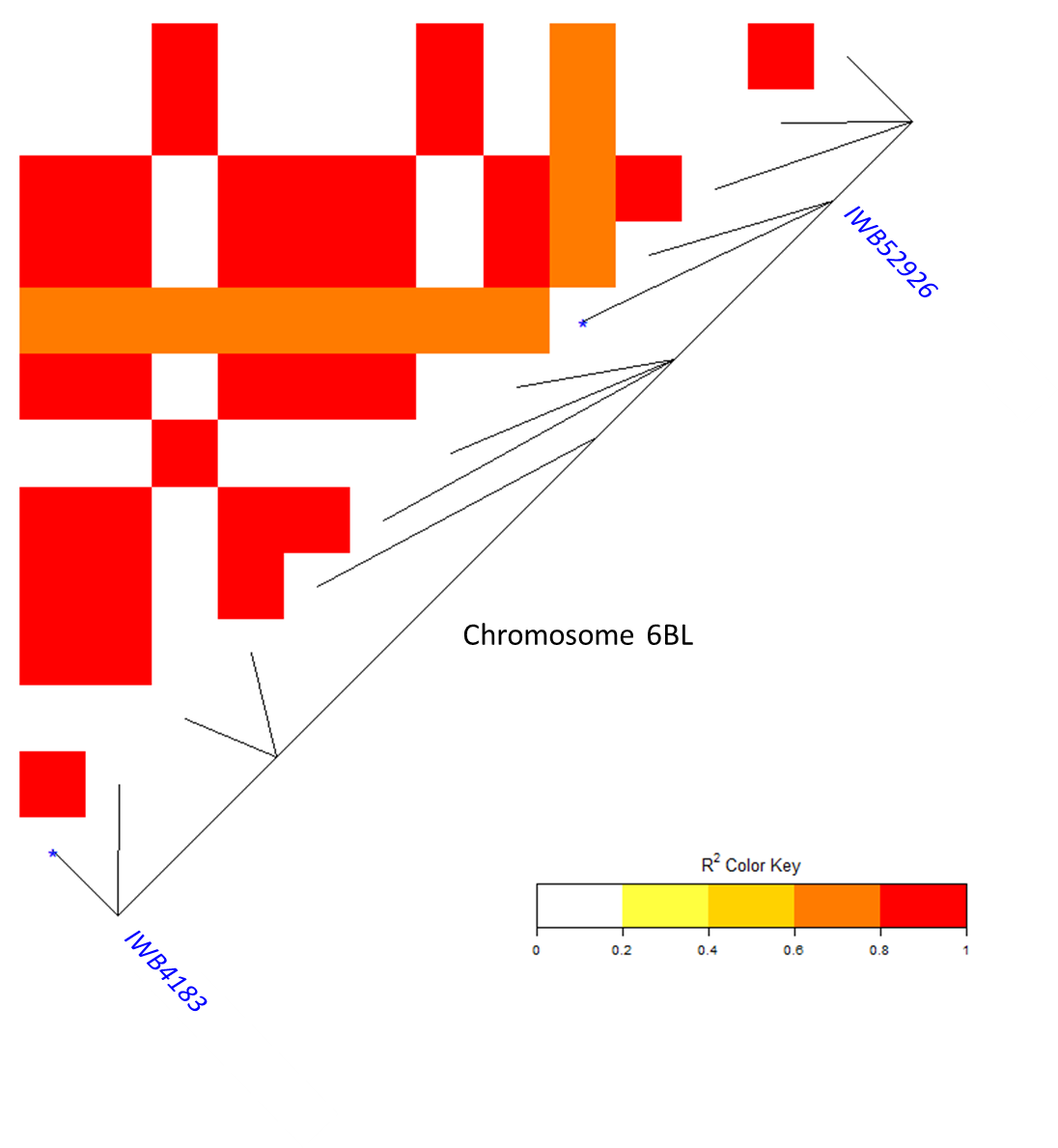
 **SUPPLEMENTARY FIGURE S3** Linkage disequilibrium (LD) heatmap between pairs of significant markers associated with leaf rust response on chromosome arms 5BS and 6BL. The color key indicates the strength of LD between markers. The markers were ordered from left to right based on their genetic positions in cM on the tetraploid consensus map of Maccaferri et al. (2015) (Supplementary Table S6).


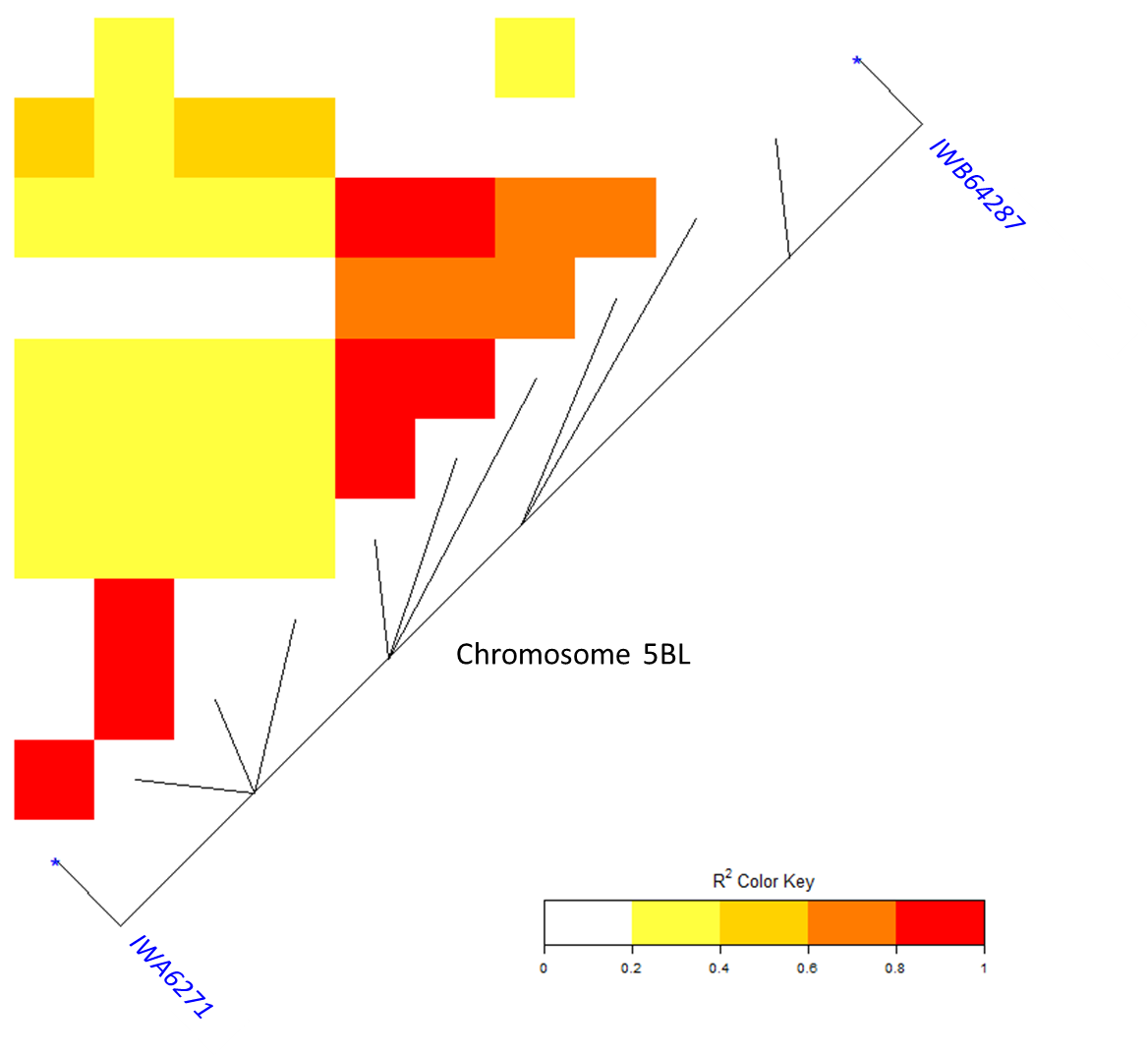


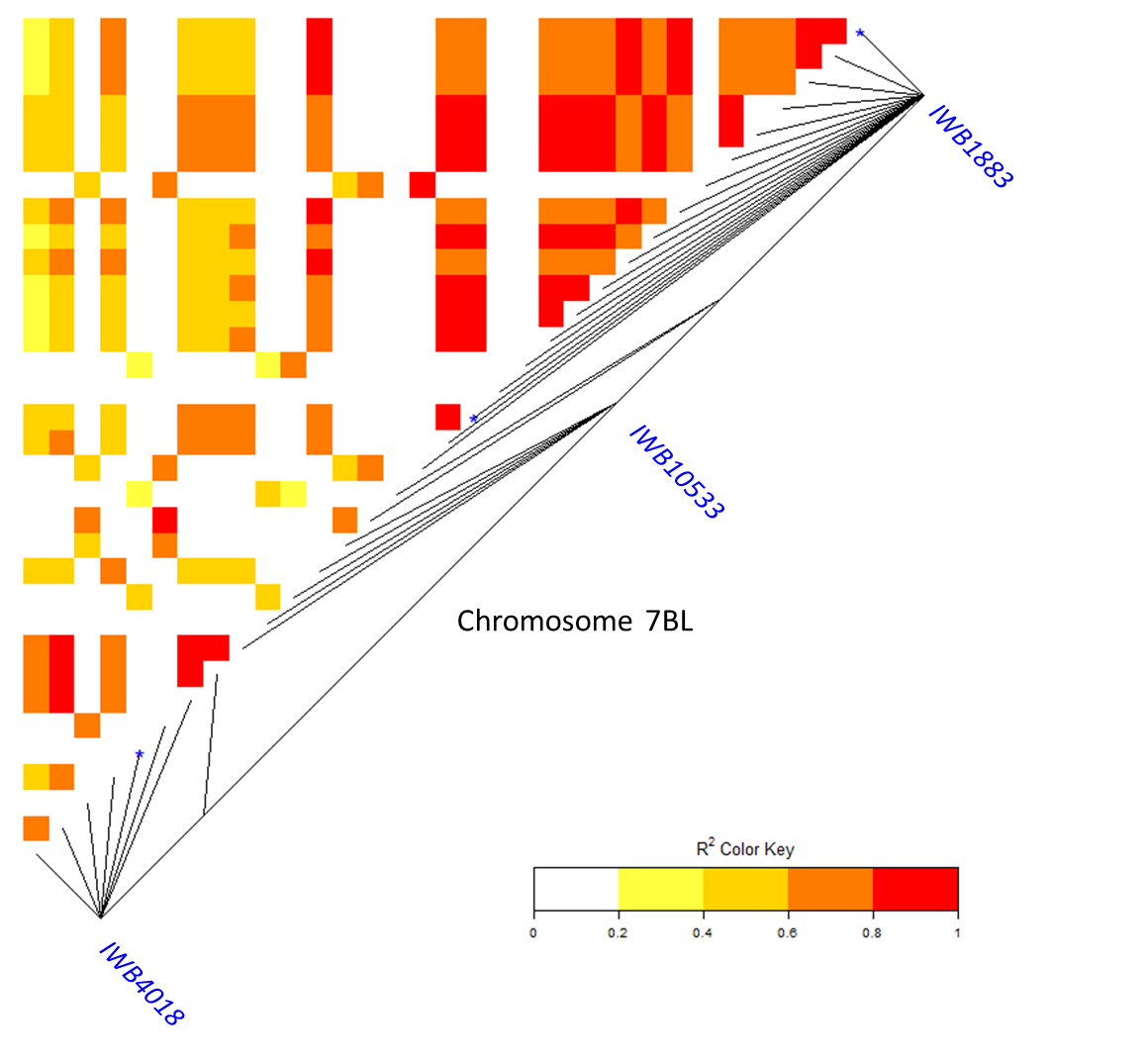


**SUPPLEMENTARY FIGURE S4** Linkage disequilibrium (LD) heatmap between pairs of significant markers associated with stripe rust response on chromosome arms 5BL and 7BL. The color key indicates the strength of LD between markers. The markers were ordered from left to right based on their genetic positions in cM on the tetraploid consensus map of Maccaferri et al. (2015) (Supplementary Table S7).


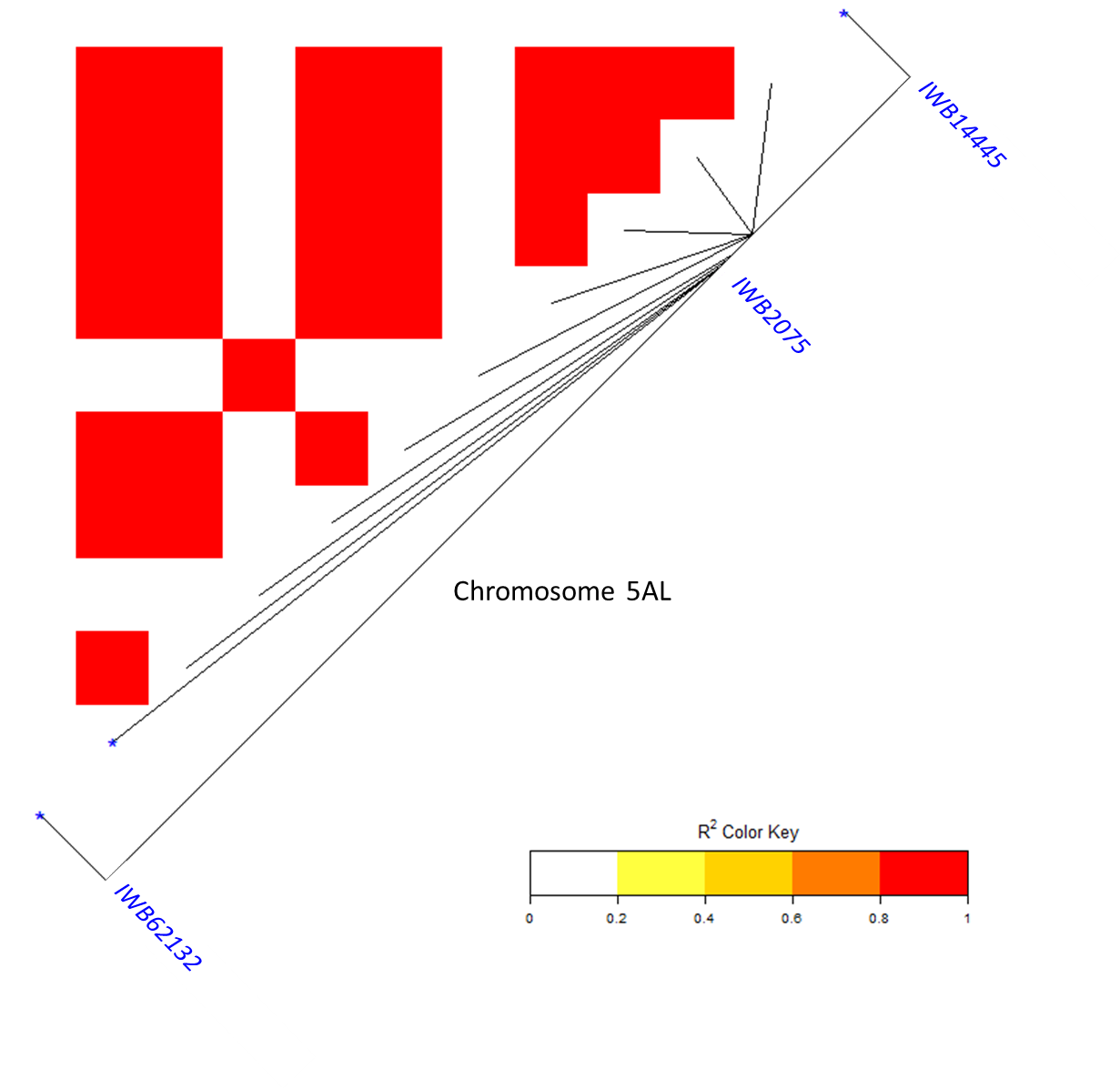


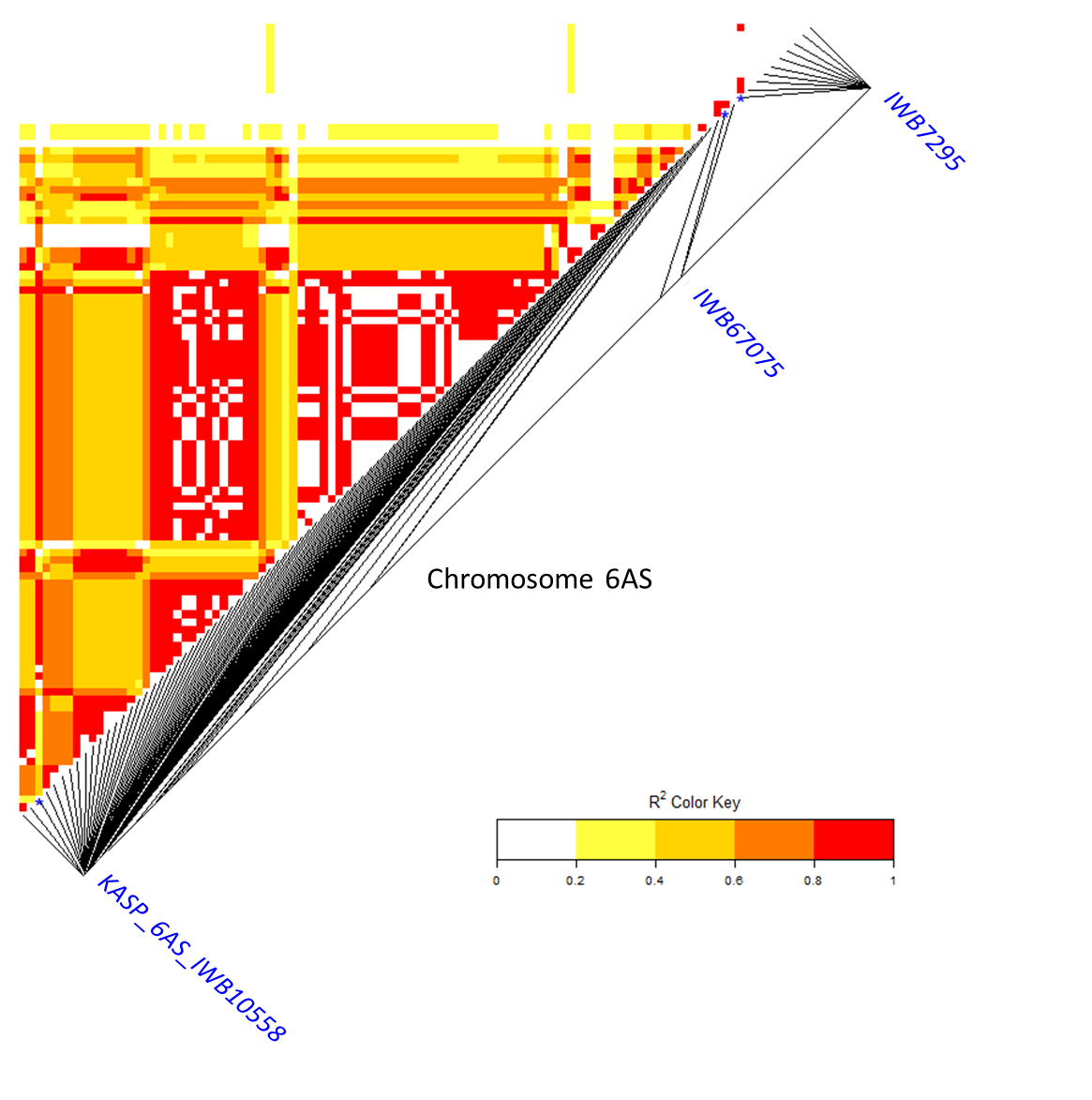


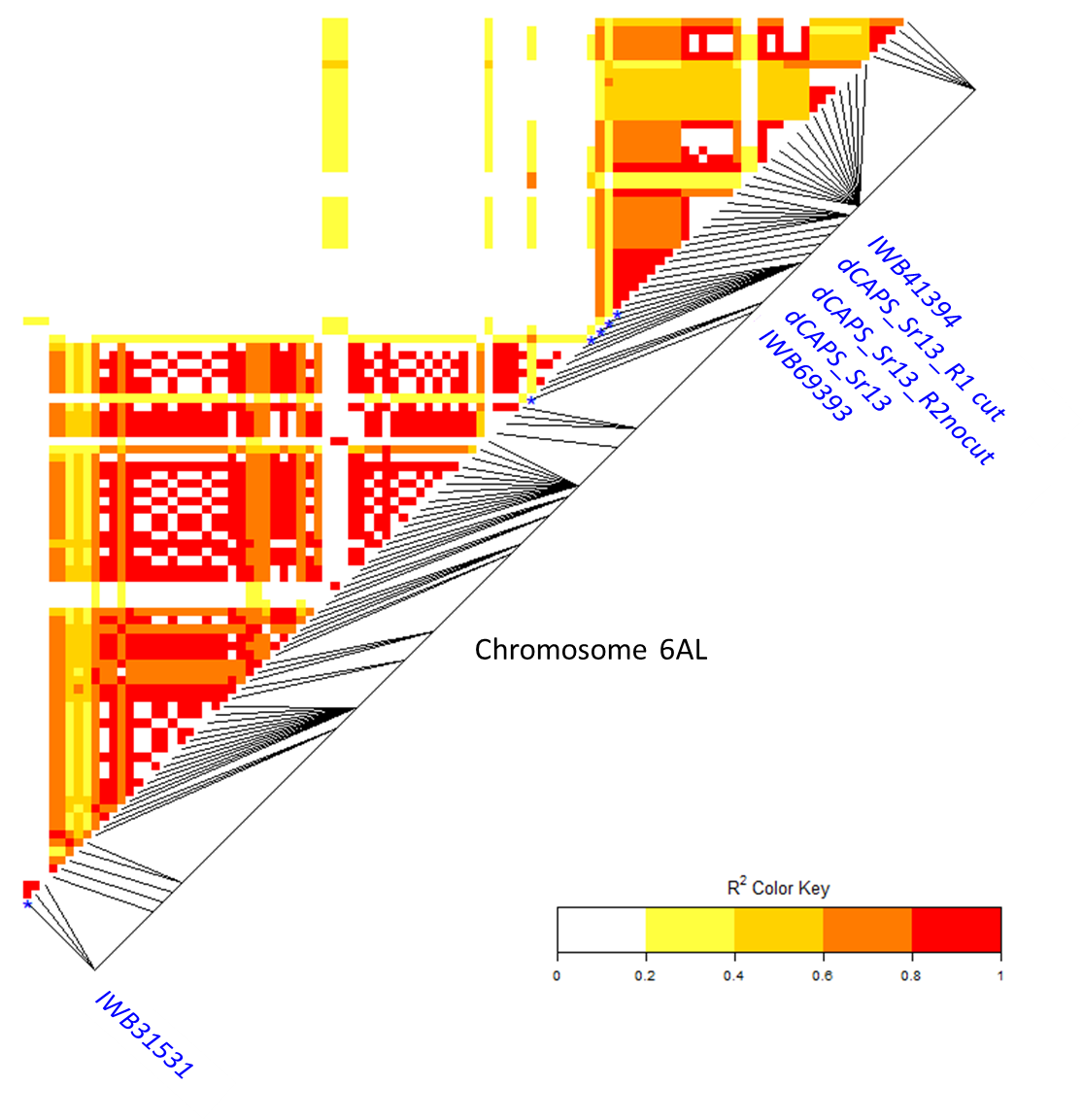


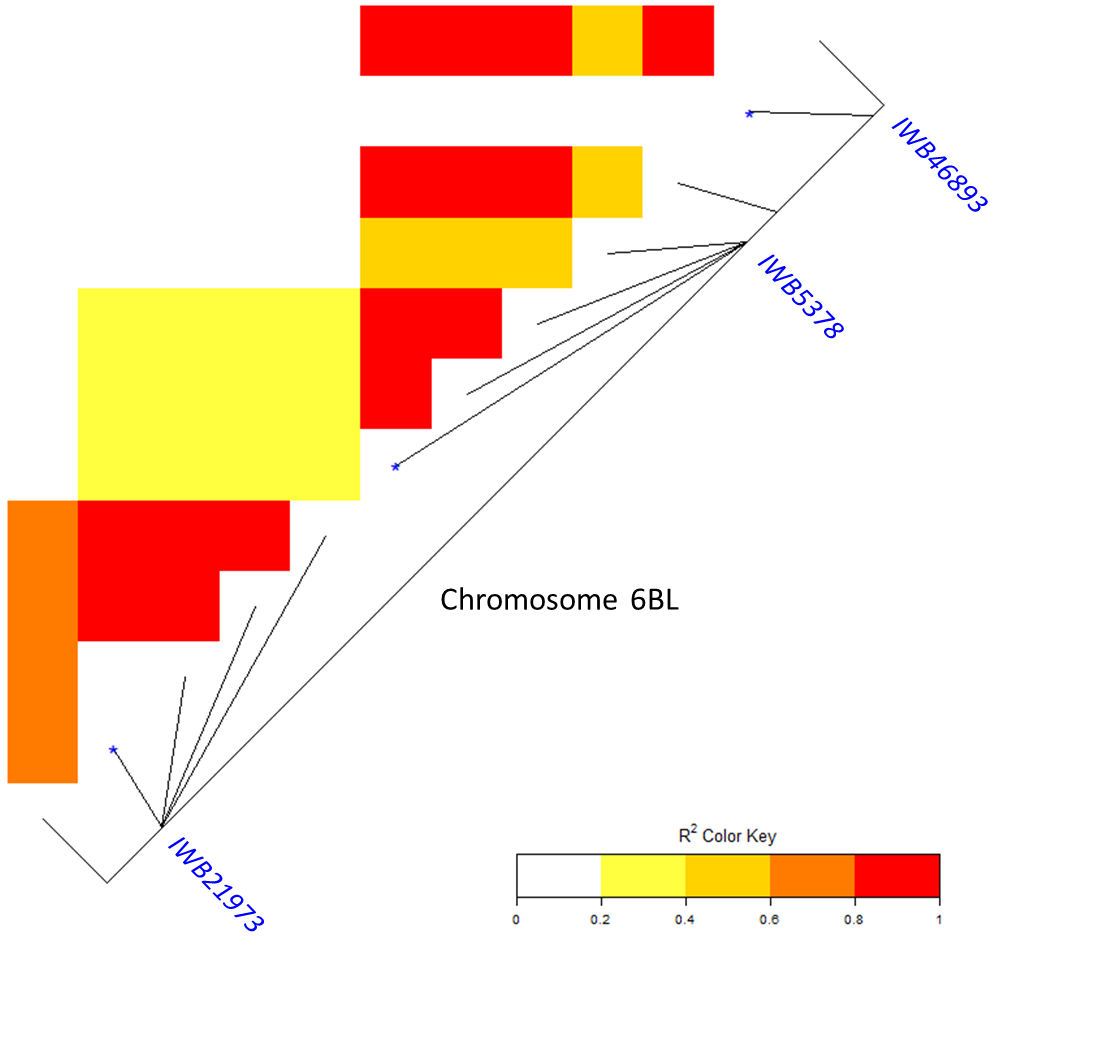


**SUPPLEMENTARY FIGURE S5**. Linkage disequilibrium (LD) heatmap between pairs of significant markers associated with stem rust response on chromosome arms 5AL, 6AS, 6AL, and 6BL. The color key indicates the strength of LD between markers. The markers were ordered from left to right based on their genetic positions in cM on the tetraploid consensus map of Maccaferri et al. (2015) (Supplementary Table S8).
